# Biogenesis of HLA Ligand Presentation in Immune Cells Upon Activation Reveals Changes in Peptide Length Preference

**DOI:** 10.3389/fimmu.2020.01981

**Published:** 2020-08-28

**Authors:** Fabio Marino, Aikaterini Semilietof, Justine Michaux, Hui-Song Pak, George Coukos, Markus Müller, Michal Bassani-Sternberg

**Affiliations:** ^1^Agora Center, Ludwig Institute for Cancer Research, University of Lausanne, Lausanne, Switzerland; ^2^Department of Oncology, Centre Hospitalier Universitaire Vaudois (CHUV), Lausanne, Switzerland; ^3^Vital IT, Swiss Institute of Bioinformatics, Lausanne, Switzerland

**Keywords:** antigen processing and presentation, dendritic cells, immunopeptidomics, mass spectrometry, cancer antigens

## Abstract

Induction of an effective tumor immunity is a complex process that includes the appropriate presentation of the tumor antigens, activation of specific T cells, and the elimination of malignant cells. Potent and efficient T cell activation is dependent on multiple factors, such as timely expression of co-stimulatory molecules, the differentiation state of professional antigen presenting cells (e.g., dendritic cells; DCs), the functionality of the antigen processing and presentation machinery (APPM), and the repertoire of HLA class I and II-bound peptides (termed immunopeptidome) presented to T cells. So far, how molecular perturbations underlying DCs maturation and differentiation affect the *in vivo* cross-presented HLA class I and II immunopeptidomes is largely unknown. Yet, this knowledge is crucial for further development of DC-based immunotherapy approaches. We applied a state-of-the-art sensitive MS-based immunopeptidomics approach to characterize the naturally presented HLA-I and -II immunopeptidomes eluted from autologous immune cells having distinct functional and biological states including CD14^+^ monocytes, immature DC (ImmDC) and mature DC (MaDC) monocyte-derived DCs and naive or activated T and B cells. We revealed a presentation of significantly longer HLA peptides upon activation that is HLA allotype specific. This was apparent in the self-peptidome upon cell activation and in the context of presentation of exogenously loaded antigens, suggesting that peptide length is an important feature with potential implications on the rational design of anti-cancer vaccines.

## Introduction

The primary aim of the adaptive immune system is to provide highly specific response against “non-self” antigens and generate memory to counter similar insults in the future (1). Professional antigen presenting cells (APCs) have the key role in presenting antigens in the context of the Human Leukocyte Antigen (HLA) complex molecules that are recognized by T cells via the T cell receptors (TCR). Intracellular degradation of proteins generates short peptides that are transferred into the Endoplasmic reticulum where they can be presented in complex with the HLA class I (HLA-I) molecules. HLA-I restricted presentation alerts the immune system about intracellular pathogens or malignant transformation by displaying targets for CD8^+^ T cells. HLA class II (HLA-II) complexes present antigens to CD4^+^ T cells. HLA-II complexes are loaded with peptides that are mainly degradation products of cellular proteins that reached the endosome-lysosome compartments (2). Dendritic cells (DCs) are uniquely equipped for taking up exogenous antigens, such as those released from dying cells in the tumor microenvironment. Exogenous antigens can then be processed within the DCs and presented on HLA-II complexes and in a process known as cross-presentation, they can be presented also on HLA class I molecules (3, 4). Due to these unique properties, DCs are the most potent primers of naïve T cells and they are essentially the master regulators of immune system (5).

Because DCs play a key role in boosting endogenous immune responses, numerous DC-based anti-cancer immunotherapies have been tested in clinical trials demonstrating that *ex vivo*-generated DCs loaded with specific tumor antigens, including mutated neoantigens, or tumor lysates, are promising therapeutic approaches (6, 7). However, so far, they have not been translated into meaningful therapeutics (8). Induction of an effective tumor immunity is a complex process that includes the appropriate presentation of the tumor antigens, activation of specific T cells, and the elimination of malignant cells (9). Tumor specific antigens are mostly self-antigens typically overexpressed or abnormally expressed in tumors. Due to the tolerance of T cells to self-antigens, much attention has been driven toward strategies to maximize the immune-stimulatory features of DCs, with the hope that these would break this tolerance (10). Importantly, potent and efficient T cell activation mediated by DCs is dependent on the DCs maturation and differentiation state, on timely expression of co-stimulatory molecules, the functionality of the antigen processing and presentation machinery (APPM) and ultimately, on the repertoire of HLA class I and II-bound peptides (termed immunopeptidome) presented to T cells (11). Several studies extensively described the cellular processes and time-dependent regulation of gene expression (12–17) mediating DCs differentiation and maturation, among them the release of cytokine and growth factors (18, 19), and the expression of co-stimulatory molecules (20) that eventually lead to their ability to process antigens and stimulate T cells (21, 22). The repertoire of the presented class I and II immunopeptidomes are constantly modulated by source protein expression levels and by several enzymes, chaperones, and transporters that comprise the APPM (23). So far, how molecular perturbations underlying DCs maturation and differentiation affect the *in vivo* cross-presented HLA class I and II immunopeptidomes is largely unknown. Furthermore, the similarity between the repertoire of HLA ligands presented on DCs and on tumor cells, and more specifically, of the epitopes presented from exogenously loaded tumor antigens, has not been thoroughly investigated. Yet, this knowledge is crucial to further develop DC-based immunotherapy approaches.

Presently, mass spectrometry (MS) is the only unbiased methodology that facilitates a comprehensive interrogation of the naturally presented HLA-bound repertoires (24). Technical limitations related to sensitivity, low throughput and reproducibility of existing methodologies posed a limit to a detailed assessment of the *in vivo* DCs-derived immunopeptidomes, due to very low starting cell amounts. In order to overcome these limitations, we recently developed an extraction pipeline for immunopeptidomics, which achieved greater sensitivity and reproducibility (23, 25). In this work, we attempted to deepen our understanding on how molecular perturbations underlying DC differentiation and maturation could affect the *in vivo* (cross-) presented HLA-I and -II immunopeptidomes. We focused on the analysis of autologous systems having distinct cell functional and biological states including CD14^+^ monocytes, immature DC (ImmDC), and mature DC (MaDC) monocyte-derived DCs, naive or activated T and B cells. We performed shotgun proteomics comparative analyses in combination with flow cytometry in order to achieve a global view on protein expression profiles, signaling pathways, and modulation of the APPM components. Uniquely to our work, we interrogated the functionality of the APPM by applying MS-based immunopeptidomics approach to characterize the naturally presented HLA-I and -II immunopeptidomes eluted from the different cell types. Upon activation and in the context of direct presentation and cross-presentation of exogenously loaded antigens, cells present significantly longer HLA peptides in HLA allotype specific manner. We discuss the importance of this feature and the potential clinical implications related to the rational design of anti-cancer vaccines.

## Materials and Methods

### Human Immune Cell Generation and Activation

Fresh peripheral blood mononuclear cells (PBMCs) were obtained from healthy donors and informed consent of the participants was obtained following requirements of the institutional review board (Ethics Commission, CHUV). The translational research has been approved by the CHUV ethics committee (protocols 2017-00305). High-resolution 4-digit HLA-I and HLA-II typing was performed for all donors at the Laboratory of Diagnostics, Service of Immunology and Allergy, CHUV, Lausanne, and provided in Supplementary Table 1.

PBMCs were separated from red blood cells (RBCs) by density gradient centrifugation with Lymphoprep and SepMate centrifuge tubes (STEMCELL Technologies SARL) by following manufacturer instructions. Monocytes CD14^+^, CD4^+^,CD8^+^, and CD19^+^ cells were separately isolated by MACS LS Columns for magnetic cell isolation with MicroBeads Human CD14^+^, CD4^+^, CD8^+^, and CD19^+^ kits, respectively (all from Miltenyi Biotec GmbH, Bergisch Gladbach, Germany), following manufacturer instructions. Purified CD14^+^ monocytes were either washed twice with ice cold PBS and stored as dry cell pellets at −20°C until use, or cultured at 1e6 cells/ml in RPMI 1640 GlutaMAX medium, with addition of 1% Penicillin/Streptomycin Solution and 10% heat-inactivated FBS. For differentiation to ImmDCs, similarly to a previously published protocol (26), CD14^+^ monocytes were cultured in the presence of recombinant research grade human granulocyte-macrophage colony stimulating factor (GM-CSF; 500 IU/ml, Miltenyi Biotec GmbH, Bergisch Gladbach, Germany) and interleukin-4 (IL-4; 250 IU/ml, Miltenyi Biotec GmbH, Bergisch Gladbach, Germany). After 2–3 days, 10% of medium was added together with fresh cytokines (GM-CSF; 500 IU/ml) and interleukin-4 (IL-4; 250 IU/ml). DCs were matured for 24 h with LPS (60 EU/ ml) and IFNγ (2,000 IU/ml, Miltenyi Biotec GmbH, Bergisch Gladbach, Germany).

For experiment comparing ImmDC and MaDC, at 6-day culture period, the growth medium of ImmDCs was doubled (5e5 cells/ ml), fresh GM-CSF and IL-4 were added (respectively, 500 and 250 IU/ml) for both cell types. For the comparison of DCs loaded with synthetic peptides, ImmDC DCs at 6-day were loaded with seven synthetic long peptides at 10 ug/ml final concentration each. ImmDCs were either kept in this culture condition for extra 24 h, or maintained in medium supplemented with LPS (60 EU/ ml) and IFNγ (2,000 IU/ml, Miltenyi Biotec GmbH, Bergisch Gladbach, Germany) for 24 h to mature into MaDC. ImmDC and MaDC DCs were collected on the same day by centrifugation at 1,200 rpm for 5 min, washed twice with ice cold PBS and stored as dry cell pellets at −20°C until use.

Similarly to a previous report (27), purified CD8^+^ and CD4^+^ cells were either washed twice with ice cold PBS and stored as dry cell pellets at −20°C until use, or cultured at 1e6 cells/ml in RPMI 1640 GlutaMAX medium, with addition of 1% Penicillin/Streptomycin Solution and 10% heat-inactivated FBS. For CD8^+^ and CD4^+^ activation, PMA (10 ng/ml) and Ionomycin (1 μg/ml) were added to the medium for 48 h. Cells were harvested, washed twice with ice cold PBS and stored as dry cell pellets at −20°C until use. Purified CD19^+^ cells were either washed twice with ice cold PBS and stored as dry cell pellets at −20°C until use, or cultured at 2e6 cells/ml in Iscove's modified Dulbecco medium (IMDM, Life Technologies, Carlsbad, CA, United States), with addition of 1% Penicillin/Streptomycin Solution and 10% heat-inactivated AB human serum. For CD19^+^ activation, IL-4 (2 ng/ml, Miltenyi Biotec GmbH, Bergisch Gladbach, Germany) and CD40L-Tri (1 μg/ml, Adipogen Life Science, Epalinges, Switzerland) were added to the medium for 48 h (28). Cells were harvested, washed twice with ice cold PBS and stored as dry cell pellets at −20°C until use.

### Flow Cytometry Analysis

Cells were phenotyped by Flow Cytometry, as indicated in the results section. Briefly, cell suspensions were incubated for 10 min at room temperature with human Fc blocking solution (BD Biosciences) to block unspecific Fc receptor binding and were subsequently stained at 4°C for 30 min with the following antibody mixes, depending on the cell type: DCs were stained with anti-human CD11c-BV711 (3.9), HLA-A,B,C-PerCP-Cy5.5 (W6/32), HLA-DR,DP,DQ- Alexa Fluor 700 (Tu39), PD-L1-BV421 (29E.2A3), PD-L2 (24F.10C12), CD40-BV605 (5C3) purchased from BioLegend, CD14-V500 (M5E2), CD80-PE-Cy7 (l307.4), CD86- Alexa Fluor 700 (2331) from BD Biosciences, and CD83-FITC (HIB15a) from Beckman Coulter. DAPI (Sigma) was added in all samples right before acquisition to determine cell viability. The following gating strategy was employed to identify them: first, viability was determined with DAPI staining and DAPI+ cells were excluded. Cells were then gated based on size and granularity (FSC/SSC) and doublets were excluded. DCs were identified by the expression of marker CD11c and lack of CD14 expression. CD19+ B cells were stained with anti-human CD19-BV711 (HIB19), HLA-A,B,C-PerCP-Cy5.5 (W6/32), HLA-DR,DP,DQ-Alexa Fluor 700 (Tu39) from BioLegend, CD80-PE-Cy7 (l307.4), CD86-Alexa Fluor 700 (2331) from BD Biosciences and CD83-FITC (HIB15a) from Beckman Coulter. Same gating strategy was used for viability, size, and doublet exclusion as with DCs and B cells were selected based on CD19 positivity. Last, CD4+ and CD8+ T cells were stained with anti-human: CD8-PE (T8), HLA-A,B,C-PerCP-Cy5.5 (W6/32), HLA-DR,DP,DQ-FITC (Tu39), PD-1-BV421 (EH12.2H7), CD38-PE-Cy7 (HIT2), CD25-APC (BC96), CCR7-APC-Cy7 (G043H7), CD69-Alexa Fluor 700 (FN50) from BioLegend, CD4-BV605 (RPA-T4) and CD137-BV711 (4B4-1) from BD Biosciences and CD54RA-ECD (2H4) from Beckman Coulter. Similar gating strategies were followed as described above, and T cells were identified based on expression of CD4 and CD8, respectively. Cells were acquired on a 5-laser LSR-SORP (BD Biosciences) and results were analyzed with FlowJo v.10 (TreeStar, California).

### Scheme of Synthetic Peptides Selection for Exogenous Dendritic Cell Loading

An in-house built database, comprising shotgun proteomics and HLA-I and HLA-II immunopeptidomics data from tumor tissues, cancer cell lines and CD14^+^, ImmDC, and MaDC was mined to select tumor antigens not expressed and/or HLA-presented in DCs (29). HLA-I and -II-bound peptides identified by MS were mapped on the sequence of the source tumor antigens and “hotspots” of antigen presentation were identified. Selected “hotspots” sequences (25 to 40 mers) were further tested with predictions for binding to the common HLA-A and -B supertype representatives. Seven long peptides were selected for synthesis and exogenous loading to ImmDC and MaDC.

### Long Synthetic Peptides Sequences

WLGVSRQLRTKAWNRQLYPEWTEAQRLDCWR (peptide from PMEL from position 32 to 61 of the protein sequence, named “PMEL32”), VLGGPVSGLSIGTGRAMLGTHTMEVTVYHRRGSR (peptide from PMEL from position 155 to 195 of the protein sequence, named “PMEL155”), SSAFTITDQVPFSVSVSQLRALDGGNKHFLRNQPL (peptide from PMEL from position 201 to 234 of the protein sequence, named “PMEL201”), YLEYRQVPGSNPARYEFLWGPRALAETSYV (peptide from MAGEA4 from position 256 to 285 of the protein sequence, named “MAGEA4”), DSKVSLQEKNCEPVVPNAPPAYEKLSAEQSPPPYSP (peptide from MLANA from position 83 to 108 of the protein sequence, named “MLANA”), DSKVSLQEKNCEPVVPNAPPAYEKLSAEQ**S**PPPYSP (peptide from MLANA from position 83 to 108 of the protein sequence, named “phoMLANA,” phosphorylation at S102 in bold), FYLAMPFATPMEAELARRSLAQDAPPL (peptide from CTAG1A or NY-ESO1 from position 90 to 116 of the protein sequence, named “CTAG1A”).

All peptides were >85% pure and purchased from Pepscan (Lelystad, The Netherlands). Peptides were resuspended in sterile pure DMSO prior loading on DCs.

### High-Throughput Purification of HLA-I and HLA-II Complexes

For high-throughput HLA-I and -II immunoaffinity purification, we used a previously described protocol from our group (23, 25). We used the 96-well single-use micro-plate with glass fiber and 10 μm polypropylene membranes (ref number: 360063, Seahorse Bioscience, North Billerica, MA). Cell lysis was performed with PBS containing 0.25% sodium deoxycholate (Sigma-Aldrich), 0.2 mM iodoacetamide (IAA) (Sigma-Aldrich), 1 mM EDTA, 1:200 Protease Inhibitors Mixture (Sigma-Aldrich), 1 mM Phenylmethylsulfonylfluoride (Roche, Basel, Switzerland), 1% octyl-beta-D glucopyranoside (Sigma-Alrich) at 4°C for 1 h. Lysis buffer was added to the cells at a concentration of 1 ml per 5e6 cells. Lysates were cleared by centrifugation with a table-top centrifuge (Eppendorf Centrifuge, Hamburg, Germany) at 4°C at 14,200 rpm for 30 min. Anti-pan HLA-I (HB95) and HLA-II (HB145) antibodies cross-linked to protein-A sepharose 4B beads (Invitrogen, Carlsbad, California) beads were loaded on their respective plates at a final bead volume of 100 ul. The lysates were loaded by gravity first through the HLA-I affinity plate and then through the HLA-II affinity plate at 4°C. The plates were washed separately using the Waters Positive Pressure-96 Processor (Waters, Milford, MA) four times with 2 ml of 150 mM sodium chloride (NaCl) (Carlo-Erba, Val de Reuil, France) in 20 mM Tris-HCl pH 8, four times with 2 ml of 400 mM NaCl in 20 mM Tris-HCl pH 8 and again four times with 2 ml of 150 mM NaCl in 20 mM Tris-HCl pH 8. Finally, the beads were washed twice with 2 ml of 20 mM Tris-HCl pH 8.

### Purification of HLA-I and HLA-II Peptides

Two Sep-Pak tC18 100 mg Sorbent 96-well plates were required for the purification and concentration of HLA-I and HLA-II peptides. Each C18 plate was handled separately. Each affinity plate was stacked on top of a C18 plate and the HLA complexes including the peptides were eluted with 500 μl of 1% TFA. The C18 wells were washed with 2 ml of 0.1% TFA. Thereafter, the HLA peptides were eluted with 500 μl of 32% acetonitrile (ACN) in 0.1% TFA. The recovered HLA-I and -II peptide samples were transferred separately into eppendorf tubes, dried using vacuum centrifugation (Concentrator plus Eppendorf) and stored at −20°C.

### Proteomics Analysis

Cell pellets of approximately 1e6 cells were resuspended in lysis buffer composed of 8 M Urea (Biochemica, Billingham, UK) and 50 mM ammonium bicarbonate (AMBIC, Sigma-Aldrich) pH 8. The cell lysates were sonicated in the Bioruptor instrument (Diagenode, B01020001, Seraing, Belgium) for 15 cycles, each cycle at maximum mA for 30 s at 4°C. Subsequently, centrifugation at 20,000 g at 4°C for 30 min separated the soluble from the insoluble protein fractions. The soluble fraction was collected and the protein concentration of the lysates was determined by a Bradford protein assay. Proteins were reduced with a final concentration of 5 mM DTT (Sigma-Aldrich) at 37°C for 60 min, followed by alkylation with a final concentration of 15 mM iodoacetamide (IAA, Sigma-Aldrich) at room temperature for 60 min in the dark. After the alkylation step, the digestion was carried out with a mixture of endoproteinase Lys-C and Trypsin (Trypsin/Lys-c Mix, Promega, Madison, WI). The first step consists of endoproteinase Lys-C digestion for 4 h at 37°C with a protein to enzyme ratio of 50:1 (w/w). Subsequently, the samples were diluted 8 times with 50 mM AMBIC to a Urea concentration of 1 M. The second step of digestion was performed with Trypsin overnight at 37°C with a substrate to enzyme ratio of 50:1 (w/w). After digestion, the samples were acidified with formic acid (FA) and desalted on C18 spin columns (Harvard Apparatus, Holliston, MA).

### LC-MS/MS Analyses

HLA-I and HLA-II peptide samples were re-suspended in 9 ul of 0.1% FA and 2/3 of the sample volume were placed in the UHPLC autosampler. For HLA-I peptidomics, the gradient of acetonitrile in 0.1% FA consisted (B) of: 0–5 min (2–5% B); 5–65 min (5–30% B); 65–70 min (30–60% B); 70–75 min (60–95% B); 75–80 min (95% B), 80–85 min (95–2% B), and 85–90 min (2% B). While for the HLA-II peptides, the gradient slope was kept the same but with a length of 60 min. For proteomics, the gradient was as such: 0–5 min (2–5% B); 5–30 min (5–9% B); 30–180 min (9–22% B); 180–230 min (22–35% B); 230–250 min (35–60% B); 250–255 min (60–95% B); 255–260 min (95% B); 260–265 min (95–5% B); and 265–270 min (5% B). All samples were acquired using the nanoflow UHPLC Easy nLC 1,200 (Thermo Fisher Scientific, LC140) coupled online to a Q Exactive HF or a HFX Orbitrap mass spectrometers (Thermo Fischer Scientific) with a nanoelectrospray ion source (Sonation, PRSO-V1, Baden-Wurttemberg, Germany). We packed the uncoated PicoTip 8 μm tip opening with 75 μm i.d. 45 cm long analytical columns with ReproSil-Pur C18 (1.9 um particles, 120 Å pore size, Dr. Maisch GmbH, Ammerbuch, Germany). Mounted analytical columns were kept at 50°C using a column oven.

For proteomics, data-dependent “top15” method was used. The mass spectrometer scan range was set to 300 to 1,650 m/z with a resolution of 60,000 (200 m/z) and the mass spectrometer scan range was set to 300–800 m/z. For MS/MS, AGC target values of 1e5 were used with a maximum injection time 25 ms at set resolution of 15,000 (200 m/z). The peptide match option was disabled. In case of unassigned precursor ion charge states or a charge state of one, no fragmentation was performed and the peptide match option was set to “preferred.” The dynamic exclusion of precursor ions from further selection was set for 20 s.

For immunopeptidomics, data was acquired with data-dependent “top10” method, which isolates within a 1.2 m/z window the ten most abundant precursor ions and fragments them by higher-energy collision dissociation (HCD) at normalized collision energy of 27%. The mass spectrometer scan range was set to 300 to 1,650 m/z with a resolution of 60,000 (200 m/z) and the AGC target value of 3e6 ions was set. For MS/MS, AGC target values of 1e5 were used with a maximum injection time of 175 ms. For HLA-I peptidomics, in case of assigned precursor ion charge states of four and above, no fragmentation was performed. For HLA-II peptidomics, in case of assigned precursor ion charge states of one, and from six and above, no fragmentation was performed.

For HLA-I experiments with DCs loaded with synthetic peptides, data was acquired twice; once with data-dependent “top10” method and once with the same method containing an inclusion list of precursors (only +1, +2, and +3 charge states), which comprised 9–12 mer peptides that were predicted by NetMHCpan4.0 (30) from the long synthetic peptides sequences (<2% rank). For the predictions, the high-resolution HLA typing of each donor was used. If predicted peptides contained methionine (M) in their sequences, then oxidized forms (+15.99491 Da) of the precursors were added to the inclusion list. In absence of target masses, the mass spectrometer was enabled to isolate and fragment other ion precursors.

### Database Search

We employed the MaxQuant computational platform version 1.5.5.1 (31) to search the peak lists against the UniProt databases (Human 42,148 entries, March 2017) and a file containing 247 frequently observed contaminants. Methionine oxidation (15.99491 Da) was set as a variable modification. For proteomics, a fixed modification of cysteine carbamidomethylation (57.02146 Da) was used. The second peptide identification option in Andromeda was enabled. A peptide spectrum match (PSM) false discovery rate (FDR) of 0.01 and no protein FDR were set for peptidomics analyses, whereas for proteomic analyses also a protein FDR of 0.01 was set. For experiments with DCs loaded with synthetic peptides, a PSM FDR of 0.05 was set and Serine/Threonine phosphorylation (79.96633 Da) was added as a variable modification due to the loading of a phosphorylated long synthetic peptide. The enzyme specificity was set as unspecific for immunopeptidomics, whereas C-terminal specificity for K and R, and maximum three miscleavages were chosen for analysis of proteomics samples. Possible sequence matches were restricted to 8 to 25 amino acids (a.a.) for immunopeptidomics and proteomics while to 8 to 30 a.a. for the experiments with DCs loaded with synthetic peptides. A maximum peptides mass of 4,600 Da for proteomics and 3,000 Da for immunopeptidomics. The initial allowed mass deviation of the precursor ion was set to 6 ppm and the maximum fragment mass deviation was set to 20 ppm. We enabled the “match between runs” option, which allows matching of identifications across different replicates in a time window of 0.5 min and an initial alignment time window of 20 min between datasets of the same donor (class I and class II separately). For proteomic analysis, “match between runs” module was enabled between all samples of the same donor and label-free quantification (LFQ) was enabled in the MaxQuant environment (32).

### Experimental Design and Statistical Rationale

A detailed description of the immunopeptidomics class I, II, and proteomics experimental design, including naming, RAW MS file names, assignment of replicates, and cell number used per experiment are, respectively, provided in Supplementary Tables 2–4. We used the Perseus computational platform version 1.5.5.3 for all statistical analysis, unless otherwise indicated (33). For class I and II immunopeptidomics data, we used the “peptides” MaxQuant output tables and Peptides matching to reverse and contaminants were filtered out and intensities were log2 transformed (Supplementary Tables 5, 6, respectively). Binding affinities to the corresponding HLA-I allotypes expressed in the donors were predicted for all 8–14 mers identified peptides using NetMHCpan4.0. The threshold for binding was set to rank 2% and the respective affinity values in nM were extracted. For length distribution analysis, peptide intensities from technical replicates of the same donor and cell type were reported as an average value. For volcano plots analysis, the intensities were normalized using “width normalization” option in Perseus and missing values were imputed by drawing random numbers from a Gaussian distribution with a standard deviation of 20% in comparison to the standard deviation of measured peptide abundances. Volcano plots of modulations in the relative intensities of HLA ligands were generated. Each dot represents a unique HLA peptide. Log2-fold changes of their abundance are indicated on the x axis and the corresponding significance levels were calculated by two-sided unpaired *t*-test with FDR of 0.01 and S0 of 1. For proteomic analysis, LFQ intensities of proteins were retrieved from the “ProteinGroups” MaxQuant output table (Supplementary Table 7), log2 transformed, and a filter was set for at least two unique peptides per protein and two valid values. Missing intensities were imputed as described above and a volcano plot was generated where the log2-fold changes of activated vs. unstimulated group are indicated on the x axis and the corresponding significance levels were calculated by two-sided unpaired *t*-test with FDR of 0.01 and S0 of 3.

## Results

### Molecular Changes Upon Differentiation and Maturation of Dendritic Cells Modulate Antigen Presentation Machinery and HLA Class I and II Presentation

**Figure 1:**
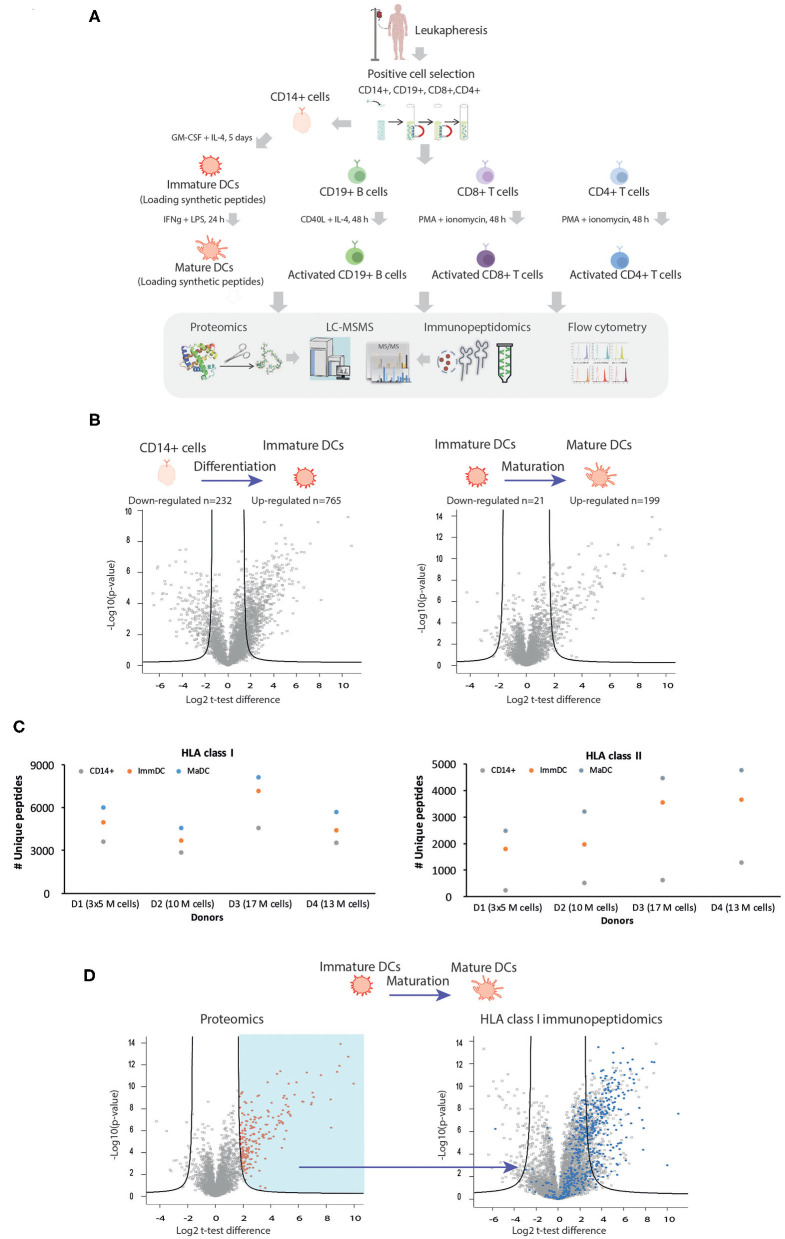
Characterization of the repertoire of HLA class I and class II ligands in different cell types with MS-based immunopeptidomics and proteomics approached. **(A)** Description of the experimental workflow. Gradient separation was used to isolate PBMCs from healthy donors' leukapheresis or apheresis samples prior to CD14^+^, CD19^+^, CD8^+^, and CD4^+^ magnetic beads positive selection. Pure CD14^+^, CD19^+^, CD8^+^, and CD4^+^ cells were either stored, or activated. In order to study the biogenesis of cross-presentation, immature, and mature DCs were in some experiments also exogenously loaded with synthetic peptides. Similar amounts of each cell population was subjected to MS-based proteomics, MS-immunopeptidomics, and flow cytometry. **(B)** Exemplary volcano plots summarizing unpaired *t*-test analysis of proteomics expression analysis upon DC differentiation (left) and maturation (right). Proteins located above the lines are statistically significantly modulated in their level of expression (FDR = 0.01, S0 = 3). **(C)** HLA-I (upper panel) and HLA-II (lower panel) uniquely identified peptides identified for CD14^+^ (blue), immature DC (orange) and mature DC (gray). **(D)** Proteome remodeling during DC maturation influences the repertoire of the HLA-I peptidome. Volcano plot (right plot) summarizing unpaired *t*-test analysis of HLA-I immunopeptidomics analysis upon DC maturation. Peptides located above the lines are statistically significantly modulated in their level of presentation (FDR = 0.01, S0= 3). The blue dots represent HLA-I peptides which belong to all source proteins found upregulated upon maturation in the proteomics analysis (red dots, left plot, FDR = 1, S0 = 3).

To characterize the biological differences occurring in DCs upon differentiation and maturation and the possible underlying changes in properties of HLA ligands naturally presented, we utilized an experimental pipeline enabling isolation and purification of sufficient amounts of CD14^+^ precursor cells from leukapheresis, apheresis filters, or buffy-coat samples (Figure 1A and Supplementary Table 1). This was an essential step for performing a range of experiments on the same samples, including comparative MS-based shotgun proteomics and immunopeptidomics analyses in combination with flow cytometry (summarized in Figure 1A and Supplementary Tables 2–7). In order to validate differentiation and maturation of DCs in each of the experimental settings, we first characterized by flow cytometry known surface makers such as HLA -I and -II complexes, PDL1, PD2L, CD40, CD80, CD83, CD86, CD11c. As previously described (34–36), we confirmed that CD14+ monocytes undergo progressive upregulation during differentiation and maturation of these markers (Supplementary Figure 1). We then evaluated global changes in protein expression and cellular processes by MS-based shotgun proteomics. Statistical analysis of proteomics expression values (Figure 1B, unpaired *t*-test) in an exemplary experiment revealed marked changes upon DC differentiation and maturation, where 199 and 765 proteins upregulated and 232 and 21 downregulated, respectively. We further focused on the expression levels of key proteins involved in HLA class I and II APPMs. As expected, upregulation of key components of the APPMs responsible for both HLA-I and HLA-II pathways was observed upon activation of CD14^+^ cells to ImmDCs and upon their differentiation into MaDCs, including CALR, CANX, TAP1/2, TAPBP, and β2m (Supplementary Figure 2). In each of the donors, gene ontology (GO) enrichment analyses (37) showed an enrichment of processes involved in antigen uptake for ImmDCs (Supplementary Table 8). ImmDC were stimulated with LPS and INFγ, and as expected, GO analysis of MaDCs revealed a strong enrichment related to immune response, cytokine-mediated signaling pathways, and cellular responses to INFs, including downstream pathways related to stimulation of TLR4 and INFγ receptors, that are known to upregulate components of the APPM (38–40) (Supplementary Table 9).

Next, we characterized with MS-based immunopeptidomics approach differences in the repertoire of naturally presented HLA-I and -II immunopeptidomes on different cellular states. Figure 1C shows enhanced antigen presentation in terms of number of unique peptides identified in both class I and II immunopeptidomes of MaDC compared to ImmDC and CD14^+^ monocytes. We identified on average 3,662, 5,067, and 6,110 HLA-I peptides from CD14^+^, ImmDC, and MaDCs, respectively, extracted from 10 to 17 million cells (Figure 1C, upper panel and Supplementary Table 5). From the same samples, we identified on average 667, 2,739, and 3,731 HLA-II peptides, respectively (Figure 1C, lower panel and Supplementary Table 6). We further assessed if changes in protein expression during maturation directly influence HLA class I peptidomic repertoire with newly synthesized and upregulated proteins being readily (within 24 h) processed and presented. We observed that source proteins significantly upregulated in MaDC were significantly more presented on HLA-I complexes (Figure 1D), supporting a direct relationship between presented class I immunopeptidomes and protein expression levels (41) and with the expression of newly synthesized proteins (42).

### Qualitative Changes in the Immunopeptidome Upon Immune Cell Stimulation

**Figure 2:**
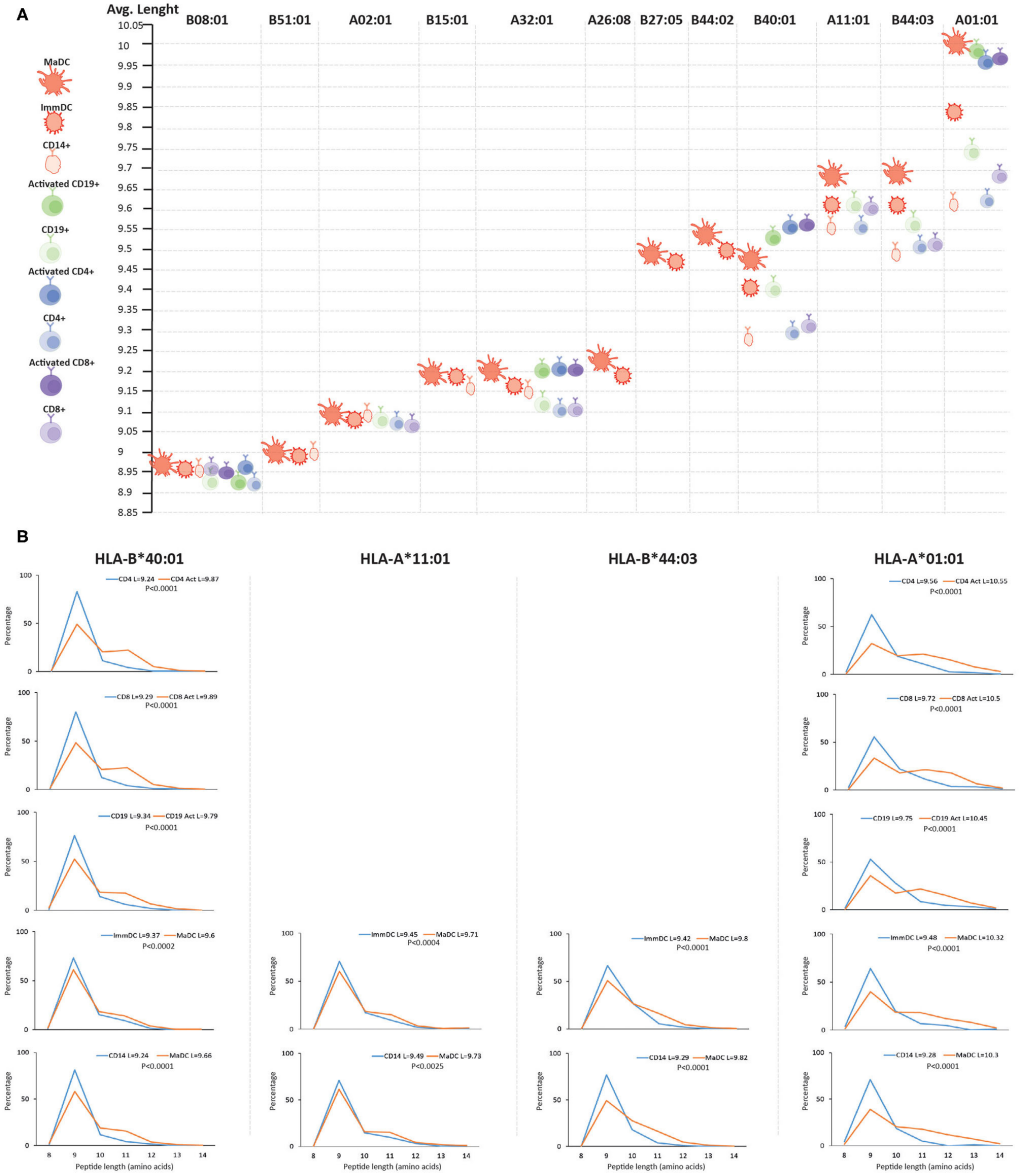
Length distribution of HLA class I peptides upon differentiation and activation of immune cells. **(A)** Length distribution across different HLA class I alleles of naive and activated immune cells. For each donor, MS detected ligands (length 8–14) were separately assigned to each of the HLA alleles with NetMHCpan 4.0 and their average length was calculated. HLA alleles are displayed from left to right based on their increasing propensity to bind longer peptides. **(B)** Length distributions (length 8–14) of unique HLA-I peptides presented in activated vs. unstimulated immune cells of HLA alleles which are expected to accommodate longer peptides. Average length (L) for each cell type is reported together with the statistical significance (unpaired *t*-test, *p*-values).

We then searched for qualitative differences in the HLA class I and II immunopeptidomes upon activation and differentiation of DCs that were mediated by changes in the APPM. First, we assigned identified peptides to the donor's HLA allotypes by predicting their binding affinity. We could detect a minor decrease in the predicted binding affinity of the peptides to their respective HLA molecules upon activation and differentiation into the MaDC state (Supplementary Figure 3). Nevertheless, we found statistically significant differences in the length of unique peptides presented by MaDC compared to ImmDC and CD14^+^ monocytes. That was the case only for HLA allotypes that have been shown to accommodate longer peptides (Figures 2A,B and Supplementary Table 10), such as HLA-B^*^40:01, HLA-A^*^11:01, HLA-B^*^44:03, and HLA-A^*^01:01 (43, 44) (*p*-values for MaDC vs. ImmDC were, respectively, 0.0002, 0.0004, 0.0001, and 0.0001, while *p*-values for MaDC vs. CD14^+^ were, respectively, 0.0001, 0.0025, 0.0001, and 0.0001). We found little or no peptide length differences in HLA allotypes that are characterized with preference for binding peptides of conserved length, such as HLA-B^*^08:01, -B^*^51:01, -A^*^02:01, and HLA-B^*^15:01 (*p*-values for MaDC vs. ImmDC were, respectively, 0.04, 0.21, 0.27, and 0.06. While *p*-values for MaDC vs. CD14+ were, respectively, 0.3, 0.6, 0.87, and 0.03). To test if this phenomenon was specific for DCs and/or dependent on TLR4 and INFγ receptors stimulation, we performed similar analyses on other immune cells before and after activation, including CD4^+^ and CD8^+^ T cells and CD19^+^ B cells, purified from the same donor (Figure 1A). We observed that upon their activation, similar modulation of the HLA processing and presentation machineries occurred, subsequently leading to upregulation of peptide presentation (Supplementary Figure 4). Alike to DCs, we found in the other tested activated immune cells a statistically significant difference in the peptide length distribution in case of HLA allotypes known to accommodate longer peptides (Figures 2A,B).

**Figure 3:**
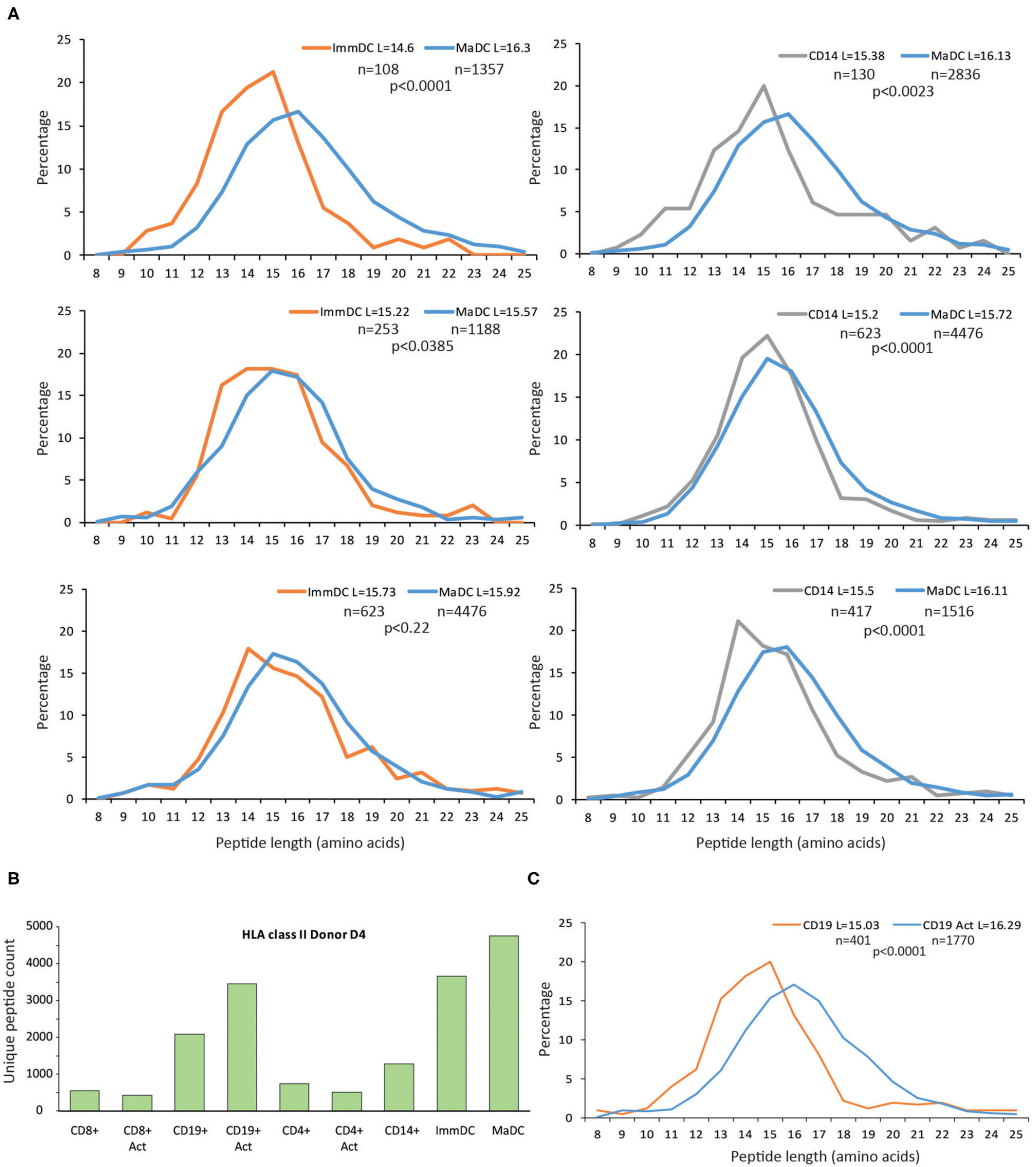
Length distribution of HLA class II peptides upon differentiation and activation of immune cells. **(A)** Upon differentiation and activation of DCs, longer peptides are presented by HLA-II allotypes. For these comparisons only donors with at least 100 unique peptides were displayed. **(B)** Overview of unique HLA class II peptides identified in CD14^+^, immature and mature DCs, CD8^+^, CD4^+^, CD19^+^ cells either activated, or unstimulated. **(C)** Length distributions of unique HLA-II peptides presented in CD19^+^ either unstimulated or activated with CD40L. Average length (L) for each cell type is reported together with the statistical significance (unpaired *t*-test, *p*-values).

Furthermore, we observed similar qualitative changes at the peptide length distribution for HLA-II peptides in DCs (Figure 3A). In general, the HLA-II immunopeptidomics data in CD4^+^ and CD8^+^ T cell samples were spares, and we could not detect substantial upregulation of HLA-II upon maturation and activation of these T cells (Figure 3B). However, in CD19^+^ cells, we observed the same qualitative changes at the HLA-II peptidomes upon activation (Figure 3C).

Distinct sets of known enzymes, chaperons, and transporters are responsible for trimming proteins into the final peptide sequences and for their loading on HLA class I and II complexes (45, 46). To unveil clues about the proteins that could possibly be responsible for the generation of longer peptides, we explored the proteomics data of the investigated cell types. However, no clear significant changes in the expression of enzymes involved in processing of HLA class I and II could support a direct link (Supplementary Figures 5, 6) with the length increase detected on the HLA-presented peptides.

### Study the Biogenesis of Cross-Presented Exogenously Loaded Antigens

**Figure 4:**
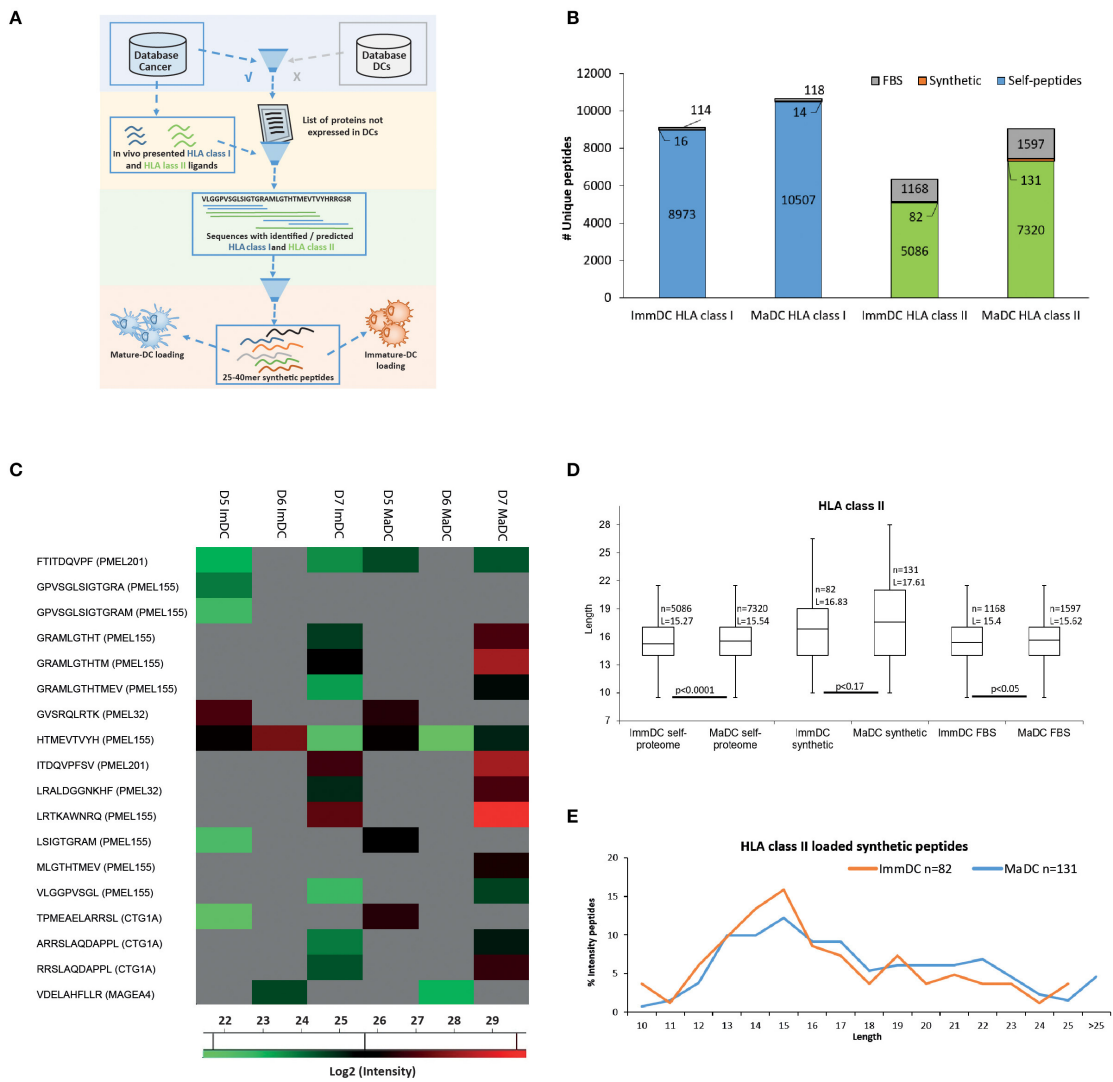
Dendritic cell exogenous loading. **(A)** Scheme of synthetic peptides selection for exogenous dendritic cell loading. In-house cancer and dendritic cell databases, respectively built by measuring protein, HLA class I and class II-bound peptide expressions from tumor tissues/cell lines and CD14^+^, immature, and mature dendritic cells. The cancer database was filtered out for proteins which were also found expressed in the dendritic cell database. HLA class I and II-bound peptides identified by MS were mapped on the remaining list of proteins. Only protein sequences which included multiple HLA class I and II identified peptides (hotspots' of antigen presentation) were further selected. Selected “hotspots” sequences (25 to 40 a.a) were further tested with predictions for HLA class I binding of the most common HLA-A and -B alleles. Seven remaining long peptides were selected for synthesis and exogenous loading to immature and mature dendritic cells. **(B)** Identified (FDR 5%) HLA class I and class II-bound FBS-derived (gray) from 3 combined donors, self- (blue for class I and green for class II) and derived from the exogenously loaded synthetic (orange) peptides from immature and mature dendritic cells. **(C)** Heat map of log2 intensity values of HLA class I peptides derived from the exogenously loaded synthetic peptides from immature and mature dendritic cells of 3 donors. **(D)** Box plot of length distributions of respectively HLA class II from cell self-proteome, derived from the exogenously loaded peptides and FBS-derived peptides are plotted for immature and mature dendritic cells of 3 donors. Average length (L) for each cell type is reported together with the statistical significance (unpaired *t*-test, *p*-values). **(E)** Intensities of HLA class II synthetic peptides identified in immature and mature dendritic cells were summed up for each peptide length and then normalized by the total intensities of identified HLA class II synthetic peptides.

DCs are important vaccine delivery vehicles in cancer immunotherapy because they can be loaded *ex vivo* with vaccine targets and present and cross-present *in vivo* the exogenously loaded antigens for priming of naïve T cells (47). Therefore, we tested if similar differences in peptide length could be observed for HLA-I and HLA-II peptides that are derived from the processing and presentation of exogenously loaded long peptides. We loaded ImmDCs and MaDCs from three donors with a mixture of equal amounts of seven long peptides (25–40 mers) from the known cancer associated antigens PMEL, MAGEA4, MLANA, and CTAG1A (NY-ESO1). The peptides used for loading were specifically selected as they contained multiple HLA class I and II peptides already identified by MS for several HLA restrictions. Importantly, the source proteins could not be identified as naturally expressed or presented in the DCs (Figure 4A). In all donors, we identified 16 and 14 presented HLA-I peptides and 82 and 131 HLA-II peptides, in ImmDC and MaDC cells, respectively (Figures 4B,C, Supplementary Tables 11, 12). Overall, upon DC maturation, overlapping cross-presented HLA-I peptides tend to be more abundant compared to ImmDCs (Figure 4C), and no significant difference in peptide length was observed between the cell types. We found that HLA class II peptides derived from the loaded synthetic peptides were on average longer compared to HLA-II peptides derived from self-proteins and from fetal bovine serum (FBS) proteins that were taken up, processed and presented by the DCs during cell culture (Figure 4D, *p*-values, respectively, 0.0001 and 0.05). HLA-II presented ligands derived from the exogenously loaded synthetic peptides were longer in MaDC than in ImmDC peptidomics samples (although not reaching a significant level). Moreover, HLA-II presented ligands derived from the exogenously loaded synthetic peptides were more abundant in MaDC than in ImmDC peptidomics samples (Figure 4E and Supplementary Figure 6).

## Discussion

While DCs play a key role in orchestrating highly complex immune responses against infection agents and tumors, the presented HLA ligandome in DCs upon their activation and maturation was largely unexplored, mainly due to the limited sensitivity and reproducibility of MS-based immunopeptidomics (24). In this study, we aimed to characterize the repertoire of naturally presented HLA-I and -II ligands in CD14^+^ precursors cells, as well as ImmDCs and MaDCs, leveraging our recently developed improved sample preparation method (23). Specifically, we searched for properties that would allow, in the future, improved prediction of immunogenic HLA ligands for clinical applications, such as for the development of personalized vaccines using DCs loaded with long peptides (48).

The cellular machineries responsible for the processing and presentation of source proteins and the presentation of their degradation products on HLA-I and -II complexes comprise multiple largely well-characterized enzymes, chaperons, and transporters. Cellular signaling pathways that are activated by inflammatory cytokines, such as INFγ, regulate their expression, and hence, differential expression of key molecules responsible for antigen processing and presentation is expected to take place during DCs differentiation and maturation. In this study we explored the extent such perturbations affect antigen presentation, by profiling modulation in protein expression and peptide presentation by shotgun proteomics and immunopeptidomics. As expected, upon DCs differentiation and maturation, we observed a marked increase in expression of multiple proteins involved in antigen presentation and an increase in cell-surface expression of both HLA-I and -II complexes, leading to a significant increase in the diversity and abundance of the presented ligands, where upregulated source proteins were found to have elevated presentation levels.

We characterized the presented peptides, and while no striking differences were detected concerning their predicted binding affinity to the respective HLA allotypes, we found that on average, HLA-I and HLA-II peptides presented in MaDCs were slightly, but significantly, longer than those presented in ImmDCs or in the CD14^+^ precursor cells. We repeatedly detected the difference in peptide length in DCs derived from multiple donors. Similarly, HLA-I and -II peptides presented in T cells and B cells upon activation were as well longer than those presented in the inactivated cell states. Here, the study was limited by the investigation of B cells and T cells from a single donor. Nevertheless, the results were consistent regardless of the cell type or the mode of activation. Importantly, the difference in peptide length was apparent only in HLA-I allotypes that can accommodate binding of longer peptides, such as in HLA-B^*^40:01, HLA-B^*^44:03, and HLA-A^*^01:01 (43). Interestingly, our results are in agreement with a recent paper by Ziegler et al. where they investigated the effect of HIV-1 infection on changes in HLA-I-presented peptides by mass spectrometry. They found a very similar trend of presentation of longer HLA-I peptides on HLA-B^*^40:01 and HLA-B^*^27:05 in CD4^+^ T cells upon activation or infection, that is HLA allotype dependent (49), where a more marked length shift was observed for HLA alleles that can accommodate longer peptides, and it was far less noticeable or not observed for more “rigid” HLA alleles, such as HLA-A^*^02:01. Importantly, we found that peptides presented on DCs from loaded long synthetic peptides follow a similar trend. The difference in peptide length was not observed for the multiple cross-presented HLA-I peptides we measured by MS, and this could be due to the fact that only a limited number of HLA-I peptides were detected in the loading experiments. However, HLA-II peptides derived from the processing of the long peptides were significantly longer in MaDCs, and in activated B cells.

One possible explanation for the presentation or detection of longer peptides following activation could be related to the relative higher expression of HLA-I allotypes that can accommodate binding of longer peptides. Higher expression of such HLA-I allotypes would lead to a higher absolute number of longer peptides, yet proportionally, the length distribution of peptides assigned per HLA-I allotype should remain constant. For example, if a given HLA-I allele binds preferentially 80% 9, 10% 10, and 10% 11 mers, a two-fold increase in its expression level will still display the same ratio (80:10:10), unless some perturbations would enforce a supply of peptide repertoire with different length characteristics, or if the supply of 9 mer peptides is the major limiting step. In a recent paper by Komov et al. IFN induced differential modulation of the HLA-A, B, and C peptidomes was investigated (50). They elegantly showed that overexpression of recombinant soluble HLA-A^*^02:01, introduced to compete for peptides with the endogenous membrane-bound HLA-A^*^02:01, did not alter the expression level or features of the presented peptidome of the membrane-bound HLA-A^*^02:01. Their results indicate that a surplus supply of peptides is available inside the Endoplasmic reticulum for loading onto the HLA-I peptide-receptive molecules, and that the availability of empty HLA molecules limits surface HLA-I expression. Therefore, the differential presentation of longer peptides in our experiments could be due to higher expression level of HLA-I allotypes, that can accommodate binding of relatively longer peptides, only if the 9 mer peptides supply is a major limiting factor.

Multiple cellular factors could be responsible for production of longer peptides, and the leading suspects are the proteases involved with peptide trimming in the endoplasmic reticulum or in the endosome-lysosome compartments, or proteins involved in the antigen-loading complex, which facilitate loading of peptides on particular HLA molecules. Activation of cells might induce cellular stress, which could consequently affect the APPM. We could not find consistent evidence to correlate differences in expression level of proteins involved in the APPMs to conclusively pinpoint the proteins responsible for the presentation of longer peptides, and in addition, their activity is likely to be regulated by other means than expression levels. Hence, the molecular mechanism leading to the presentation of longer peptides upon cellular activation and maturation is still unknown, and more detailed research is required for resolving this.

The similarity between the self-peptidome presented on cancer cells and on autologous antigen presenting cells is still largely unknown. Hence, our ability to select cancer-specific antigens for DC-based vaccines (e.g., synthetic long peptides and RNA vaccines), that are naturally presented with identical epitopes on both loaded DCs and on the autologous tumor cells *in vivo*, is limited. Overall, the notion is that antigen presentation is similar in both cell types. Here we provide first evidence that the presentation of HLA ligands in DCs is a dynamic process and we report that longer peptides are presented on DCs upon maturation, in an HLA-dependent manner. Ziegler et al. recently demonstrated that HIV-1 viral infection of stimulated CD4^+^ T-cells induced a significant shift toward presentation of longer HLA-I peptides (49) and several years ago it has been shown by Yaciuk et al. that epitopes as long as 20 amino acids were eluted from HLA-A^*^11:01 HIV-1 infected CD4^+^ T cells (51). In addition, McMurtrey et al. showed that in cells infected with Toxoplasma gondii, HLA-I peptides derived from the pathogen were longer compared to the baseline ligandome derived from the host proteome (52). Furthermore, highly immunogenic bulged long HLA-I binding peptides, for example from HIV, cytomegalovirus and EBV, have been shown to interact strongly with TCRs (51, 53–57). In a previous study we showed that tumor cells in the presence of inflammatory cytokine, like INFγ, present longer HLA-I peptides (23), suggesting a common mechanism enabling presentation of similar epitopes in both the antigen presenting cells and the inflamed diseased cells, that are distinctly different (by length) than ubiquitous “healthy” self-peptidome. We hypothesize that in the tumor microenvironment, activated DCs will efficiently process and present a larger fraction of longer peptides, for example from tumor antigens released from dying tumor cells, and hence, will prime T cells also against these longer epitopes, that will be also presented directly on the tumor cells in the inflamed microenvironment (49, 51–57). We envision that including such features in immunogenicity prediction tools would improve their efficacy and the development of cancer vaccines and T cell based immunotherapies. More research however, is required to further decipher the cellular mechanisms that are directly involved in shaping the immunopeptidome in the tumor microenvironment as well as under chronic exposure to inflammatory signals.

## Data Availability Statement

MS raw files, MaxQuant proteomics and immunopeptidomics output results have been deposited to the ProteomeXchange Consortium via the PRIDE (58) partner repository with the dataset identifier PXD020011.

## Ethics Statement

The studies involving human participants were reviewed and approved by the CHUV ethics committee (protocols 2017-00305). The patients/participants provided their written informed consent to participate in this study.

## Author Contributions

FM and MB-S conceived and planned the experiments. FM and AS carried out the experiments. JM contributed to sample preparation. H-SP contributed to mass spectrometry analyses. MM contributed to bioinformatics analyses. GC supported with interpretation of the results. FM and MB-S took the lead in writing the manuscript. All authors provided critical feedback, helped shape the research, analysis, and manuscript, contributed to the article, and approved the submitted version.

## Conflict of Interest

The authors declare that the research was conducted in the absence of any commercial or financial relationships that could be construed as a potential conflict of interest.

## Supplementary Material

Supplementary Figure 1 Representative FACS data of DC maturation and T cell activation marker expression. **(A)** Upon maturation with IFNg and LPS, HLA expression is upregulated, as well as a number of other markers. Cells were gated as following: Upon dead, size, and doublet exclusion, DCs were identified as CD11c^+^ and CD14^−^. **(B)** PMA/Ionomycin stimulation of T cells from the same donor as in A, results in upregulation of various activation markers. As with DCs, DAPI+ dead cells, and doublets were excluded and CD8^+^ and CD4^+^ were subsequently selected.

Supplementary Figure 2 Overview of donor D1 HLA class I presentation pathway. Log2 fold change of protein LFQs values between immature and mature DCs of proteins involved in HLA- I antigen presentation pathway in donor D1.

Supplementary Figure 3 Overview of donor D1 HLA class I presentation pathway. Box plot of affinities (nM) predicted with NetMHCpan 4.0 of CD14^+^, immature and mature dendritic cells. Distribution of predicted affinities for each cell type is reported together with the statistical significance (unpaired *t*-test, *p*-values).

Supplementary Figure 4 Overview of donor D4 HLA class I presentation in all immune cell types activated or unstimulated. **(A)** Log2 fold change of protein LFQs between activated and unstimulated immune cells of proteins involved in HLA-I antigen presentation pathway in donor D4. **(B)** number of unique HLA-I peptides identified in CD14^+^, immature and mature DCs, CD8^+^, CD4^+^, CD19^+^ cells either activated, or unstimulated in donor D4.

Supplementary Figure 5 Proteomics expression of processing enzymes possibly involved in HLA- I pathway. **(A)** Log2 fold change of protein LFQs between mature and immature DCs of enzymes possibly involved in HLA-I antigen processing pathway. **(B)** Log2 fold change of protein LFQs between activated and unstimulated immune cells from the same donor (D4) of enzymes possibly involved in HLA- I antigen processing pathway.

Supplementary Figure 6 Proteomics expression of processing enzymes possibly involved in HLA class II pathway. Log2 fold change of protein LFQs between mature and immature dendritic cells of enzymes possibly involved in HLA class II antigen processing pathway.

Supplementary Figure 7 Log2 intensity values of HLA-II peptides identified in immature and mature DCs from 3 donors derived from the exogenously loaded synthetic peptides PMEL 155-195 and PMEL 201-234.

Supplementary Table 1 Donors and experimental details. For each donor used, specimen type, HLA class I typing, immune cell type extracted, and type of experiments executed per cell type are described.

Supplementary Table 2 Experimental design HLA class I immunopeptidomics. For each donor's HLA class I experiments, cell types, experiment type, replicate type, amount of cell used, and mass spectrometry RAW file names are listed.

Supplementary Table 3 Experimental design HLA class II immunopeptidomics. For each donor's HLA class II experiments, cell types, experiment type, replicate type, amount of cell used, and mass spectrometry RAW file names are listed.

Supplementary Table 4 Experimental design proteomics. For each donor's proteomics experiments, cell types, experiment type, replicate type, amount of cell used, and mass spectrometry RAW file names are listed.

Supplementary Table 5 MaxQuant Peptide output table of Donor 1 to 4 HLA class I identified peptides.

Supplementary Table 6 MaxQuant Peptide output table of Donor 1 to 4 HLA class II identified peptides.

Supplementary Table 7 MaxQuant Protein group output table of Donor 1 to 4 proteomics experiments.

Supplementary Table 8 GO enrichment of molecular functions upon DC differentiation. GO enrichment of molecular functions was calculated by String (version 10.5). For this analysis, from each donor were used proteins found to be significantly upregulated (FDR = 1%, S0 = 3) upon monocyte differentiation to immature dendritic cells in proteomics analysis. The top 20 enriched functions are reported.

Supplementary Table 9 GO enrichment of molecular functions upon DC maturation. GO enrichment of molecular functions was calculated with String (version 10.5). For this analysis, from each donor were used proteins found to be significantly upregulated (FDR = 1%, S0 = 3) upon maturation of immature dendritic cells in proteomics analysis. The top 20 enriched functions are reported.

Supplementary Table 10 Average peptide length across different HLA class I alleles of naive and activated immune cells (related to Figure 2A). For each donor, MS detected ligands (length 8–14) were separately assigned to each of the HLA alleles with NetMHCpan 4.0 and their average length was calculated.

Supplementary Table 11 HLA class I peptides from synthetic peptides from Donor 5 to 7.

Supplementary Table 12 HLA class II peptides from synthetic peptides from Donor 5 to 7.
