# Supplementary figures and images for: Biogenesis of HLA Ligand Presentation in Immune Cells Upon Activation Reveals Changes in Peptide Length Preference
Source: Front Immunol. 2020 Aug 28;11:1981. doi: 10.3389/fimmu.2020.01981 (PMC7485268; doi:10.3389/fimmu.2020.01981)

Supplementary Figure 1

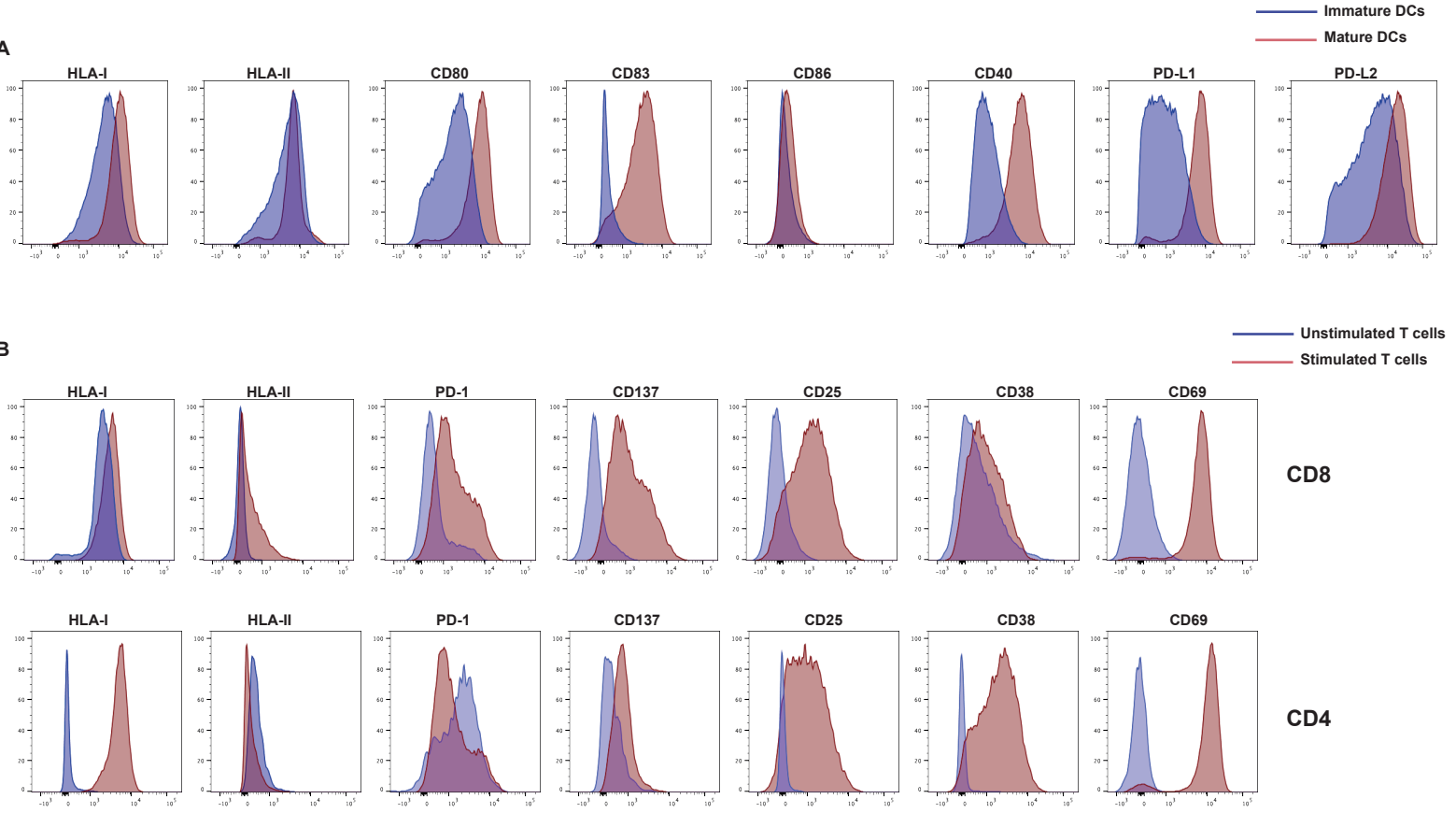

Supplement: Supplementary Figure 1 — Representative FACS data of DC maturation and T cell activation marker expression. (A) Upon maturation with IFNg and LPS, HLA expression is upregulated, as well as a number of other markers. Cells were gated as following: Upon dead, size, and doublet exclusion, DCs were identified as CD11c+ and CD14−. (B) PMA/Ionomycin stimulation of T cells from the same donor as in A, results in upregulation of various activation markers. As with DCs, DAPI+ dead cells, and doublets were excluded and CD8+ and CD4+ were subsequently selected. [file Image_1.PDF]

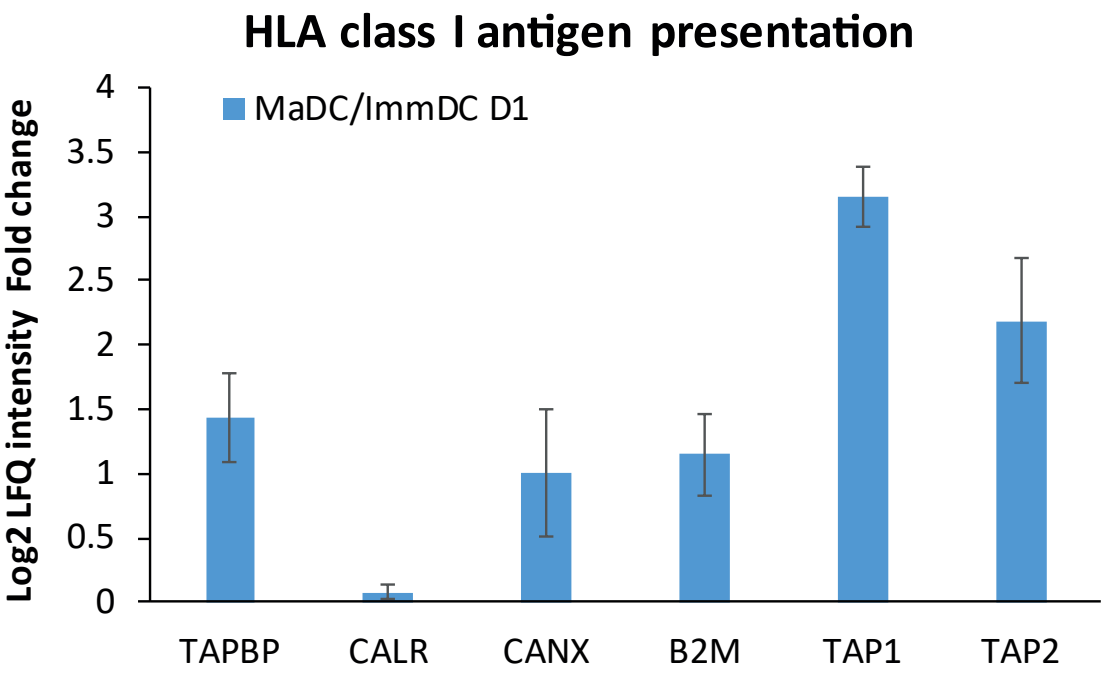

Supplement: Supplementary Figure 2 — Overview of donor D1 HLA class I presentation pathway. Log2 fold change of protein LFQs values between immature and mature DCs of proteins involved in HLA- I antigen presentation pathway in donor D1. [file Image_2.PDF]

Supplementary Figure 3

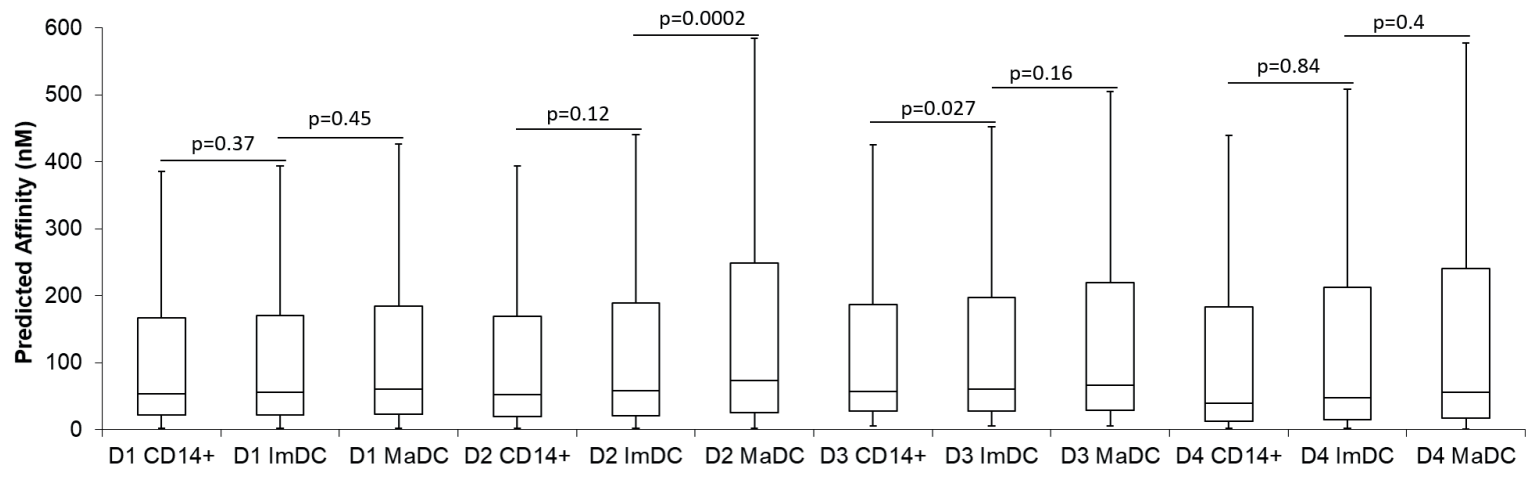

Supplement: Supplementary Figure 3 — Overview of donor D1 HLA class I presentation pathway. Box plot of affinities (nM) predicted with NetMHCpan 4.0 of CD14+, immature and mature dendritic cells. Distribution of predicted affinities for each cell type is reported together with the statistical significance (unpaired t-test, p-values). [file Image_3.PDF]

A

HLA class I antigen presentation

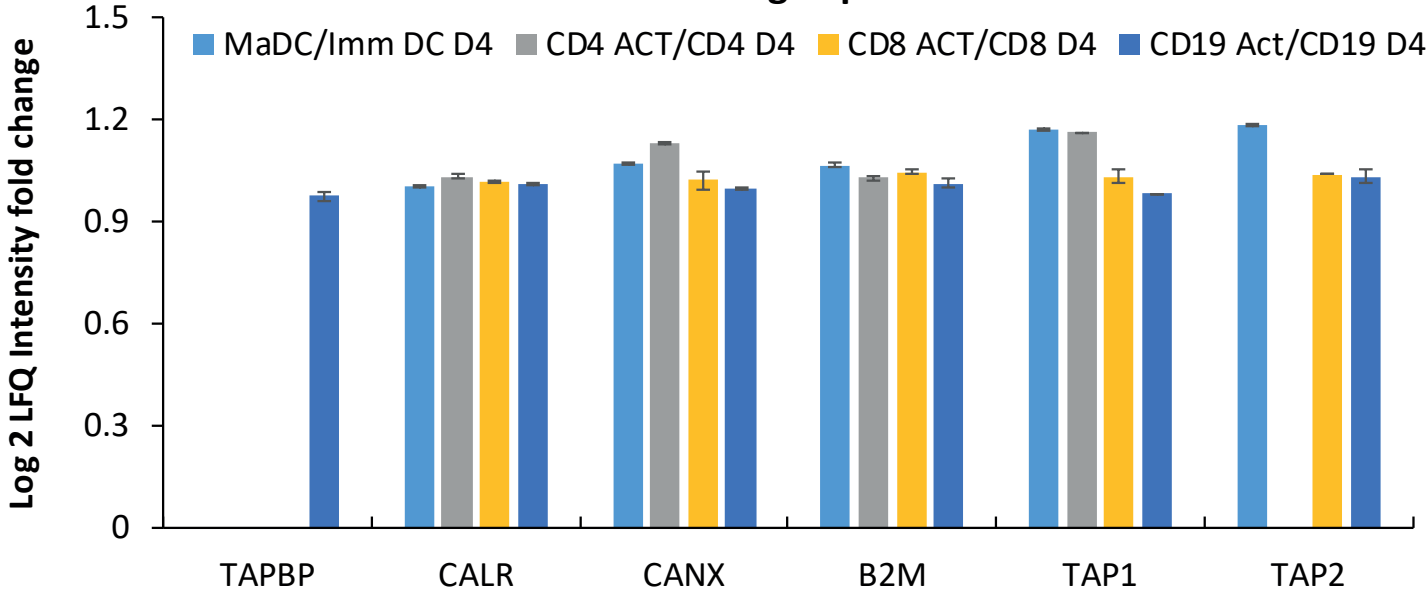

B

HLA class I Donor D4

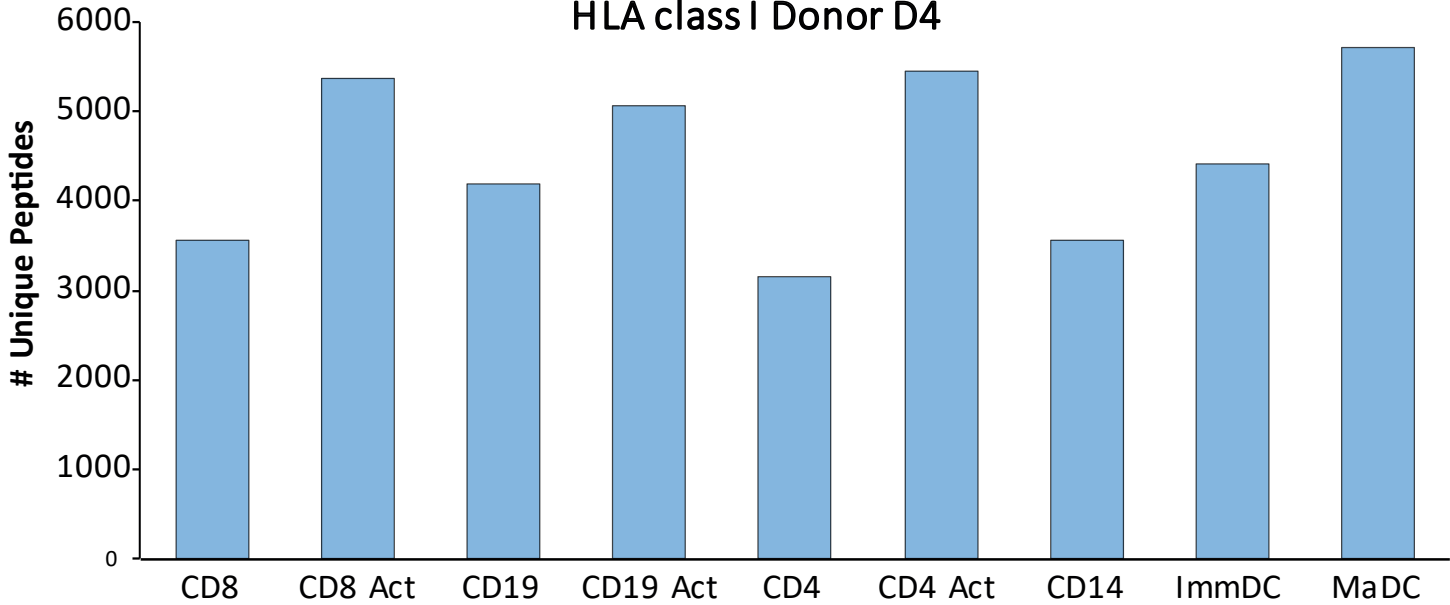

Supplement: Supplementary Figure 4 — Overview of donor D4 HLA class I presentation in all immune cell types activated or unstimulated. (A) Log2 fold change of protein LFQs between activated and unstimulated immune cells of proteins involved in HLA-I antigen presentation pathway in donor D4. (B) number of unique HLA-I peptides identified in CD14+, immature and mature DCs, CD8+, CD4+, CD19+ cells either activated, or unstimulated in donor D4. [file Image_4.PDF]

A

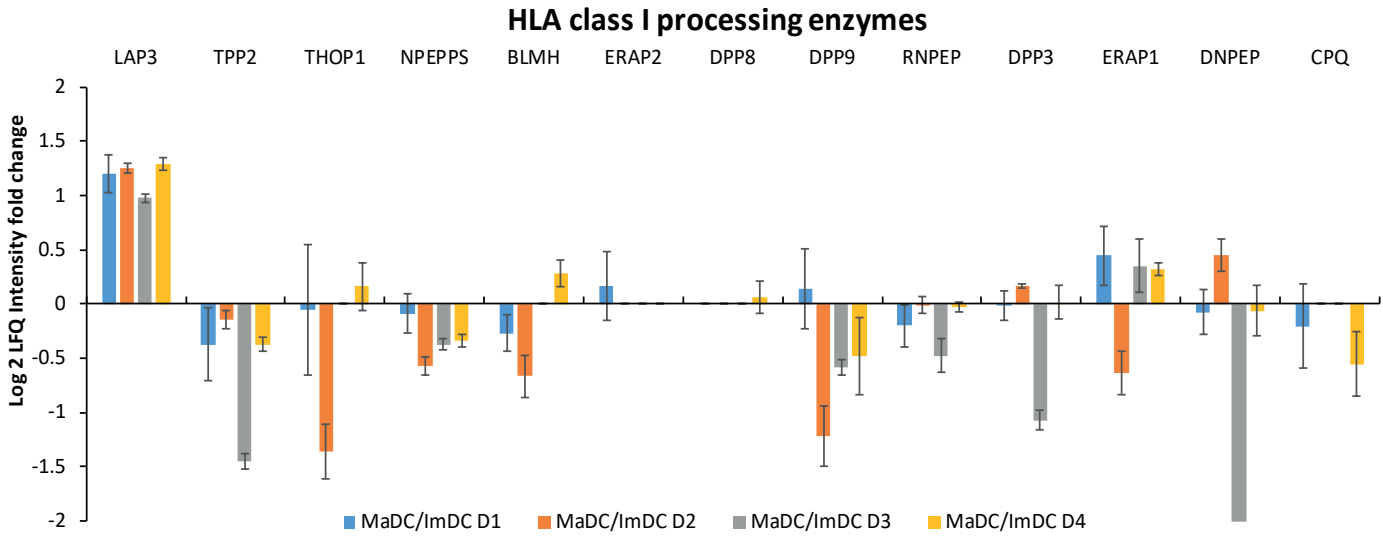

B

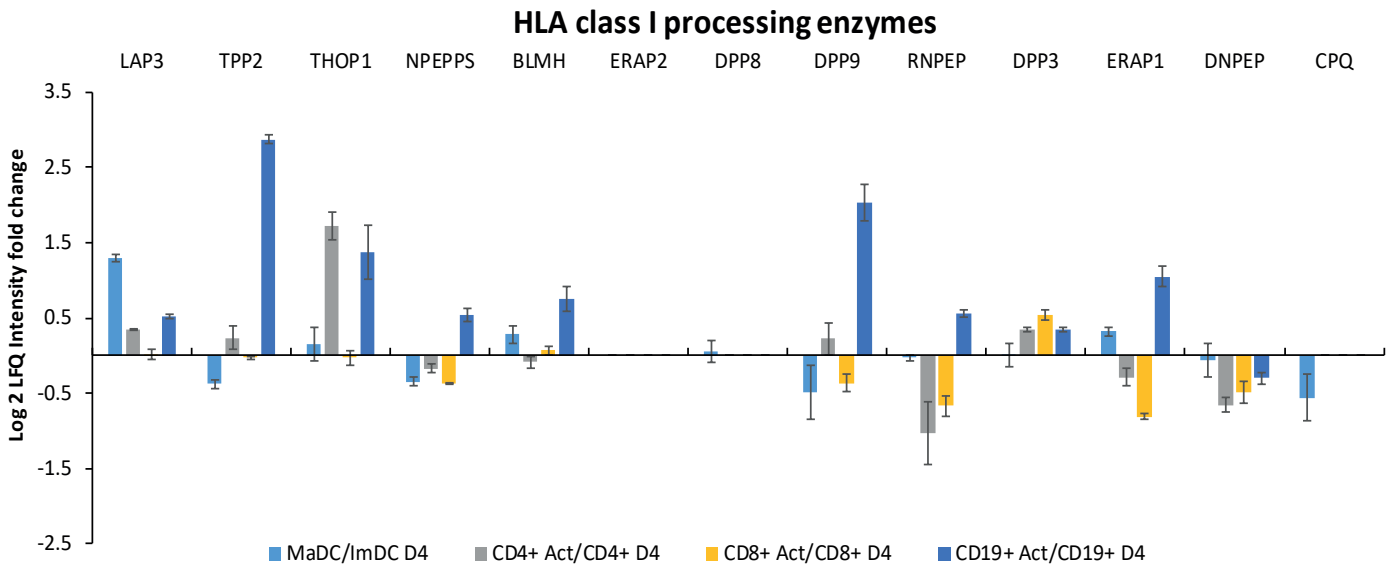

Supplement: Supplementary Figure 5 — Proteomics expression of processing enzymes possibly involved in HLA- I pathway. (A) Log2 fold change of protein LFQs between mature and immature DCs of enzymes possibly involved in HLA-I antigen processing pathway. (B) Log2 fold change of protein LFQs between activated and unstimulated immune cells from the same donor (D4) of enzymes possibly involved in HLA- I antigen processing pathway. [file Image_5.PDF]

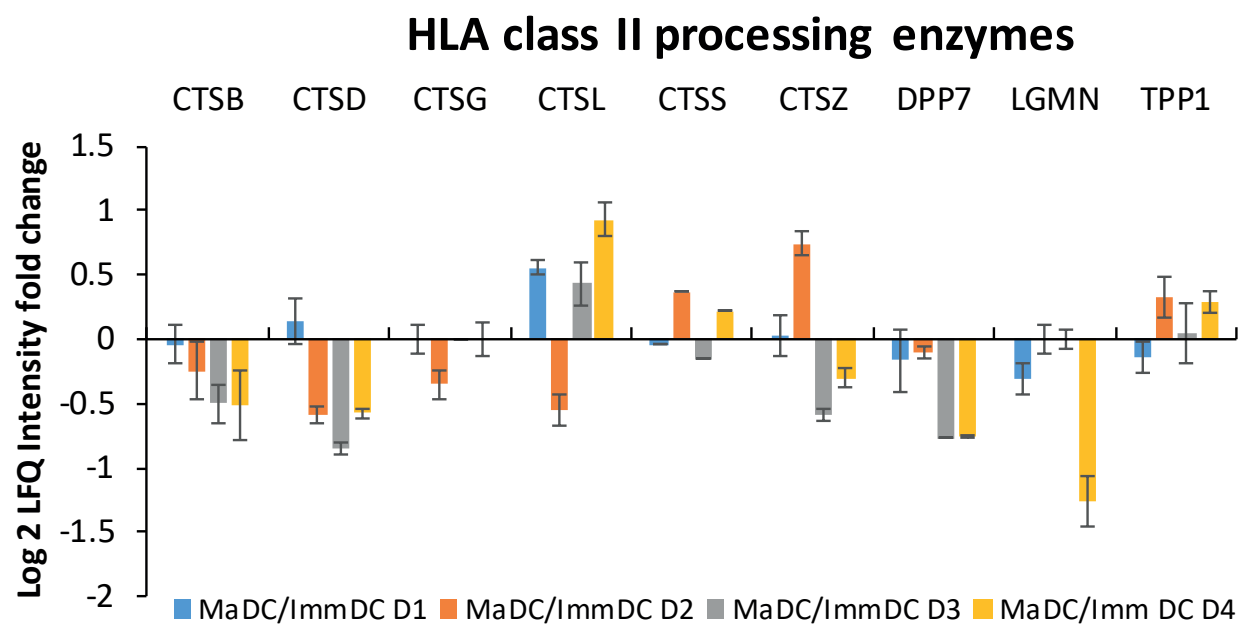

Supplement: Supplementary Figure 6 — Proteomics expression of processing enzymes possibly involved in HLA class II pathway. Log2 fold change of protein LFQs between mature and immature dendritic cells of enzymes possibly involved in HLA class II antigen processing pathway. [file Image_6.PDF]

Supplementary Figure 7

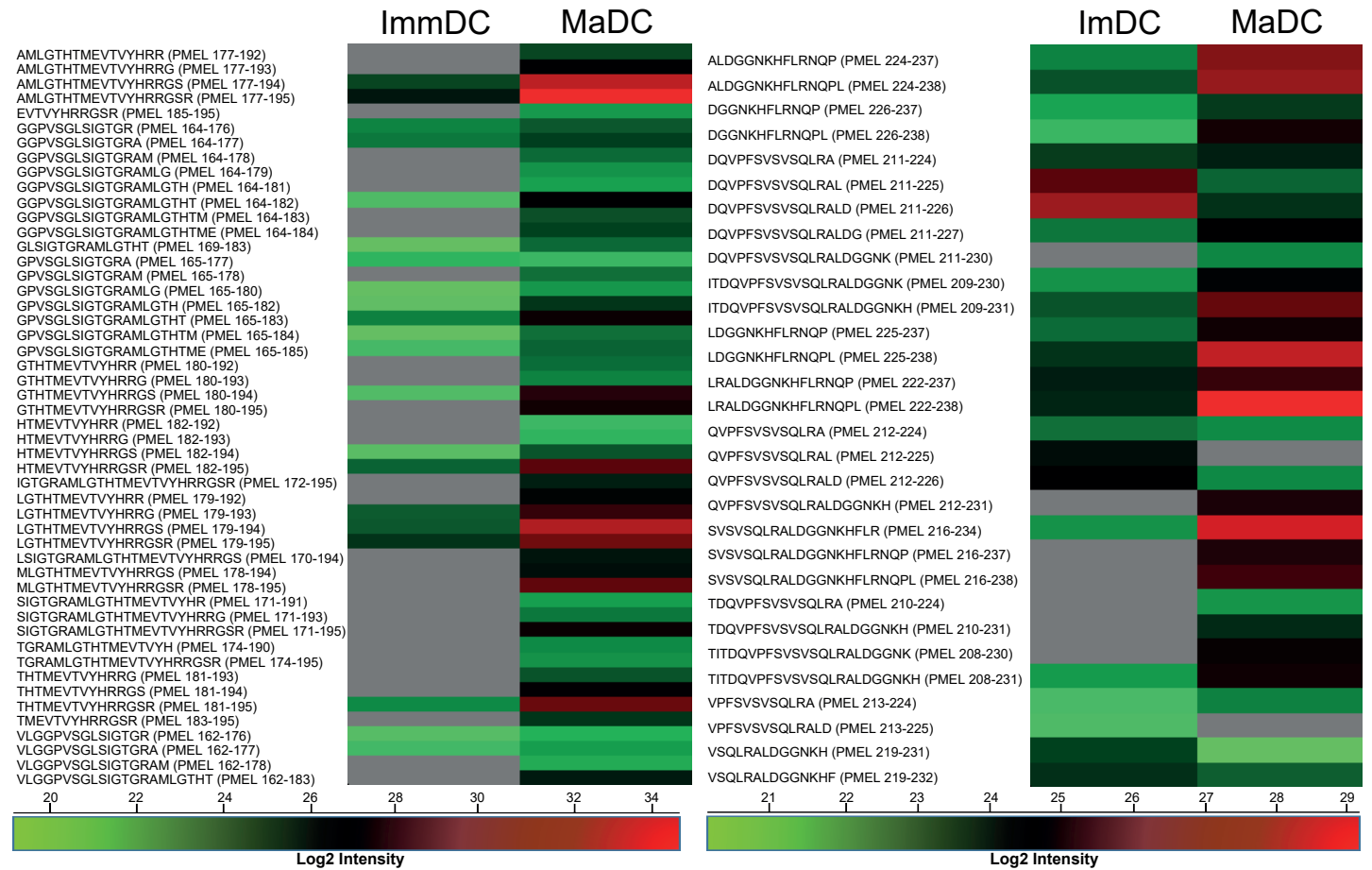

Supplement: Supplementary Figure 7 — Log2 intensity values of HLA-II peptides identified in immature and mature DCs from 3 donors derived from the exogenously loaded synthetic peptides PMEL 155-195 and PMEL 201-234. [file Image_7.PDF]
