# Supplementary material for: Biogenesis of HLA Ligand Presentation in Immune Cells Upon Activation Reveals Changes in Peptide Length Preference
Source: Front Immunol. 2020 Aug 28;11:1981. doi: 10.3389/fimmu.2020.01981 (PMC7485268; doi:10.3389/fimmu.2020.01981)
Supplement: Supplementary Table 1 — Donors and experimental details. For each donor used, specimen type, HLA class I typing, immune cell type extracted, and type of experiments executed per cell type are described. [file Data_Sheet_1.PDF]

Supplementary Table 1

**Donor name**	**Sample type**	**HLA-A**	**HLA-A**	**HLA-B**	**HLA-B**	**HLA-C**	**HLA-C**	**HLA-DQB1**	**HLA-DQB1**	**HLA-DRB1**	**HLA-DRB1**
		1st allele	2nd allele	1st allele	2nd allele	1st allele	2nd allele	1st allele	2nd allele	1st allele	2nd allele
**D1**	Leukapheresis	A:02:01	A:11:01	B:15:01	B:51:01	C:03:04	C:14:02	DQB1:03:03	DQB1:04:02	DRB1:08:01	DRB1:09:01
**D2**	Apheresis filter	A:02:01	x	B:40:01	B:44:03	C:02:02	C:03:04	DQB1:02:02	DQB1:06:02	DRB1:07:01	DRB1:15:01
**D3**	Apheresis filter	A:01:01	A:11:01	B:08:01	B:44:03	C:04:01	C:07:21	DQB1:02:01	DQB1:02:02	DRB1:03:01	DRB1:07:01
**D4**	Leukapheresis	A:01:01	A:32:01	B:08:01	B:40:01	C:03:04	C:07:01	DQB1:02:01	DQB1:05:01	DRB1:01:01	DRB1:03:01
**D5**	Buffy coat	A:03:01	A:26:01	B:07:02	B:15:01	C:03:03	C:07:02	DQB1:03:01	DQB1:06:02	DRB1:11:03	DRB1:15:01
**D6**	Leukapheresis	A:01:01	A:03:01	B:51:01	X	C:15:02	X	DQB1:03:03	DQB1:06:03	DRB1:09:01	DRB1:13:01
**D7**	Buffy coat	A:26:08	A:32:01	B:27:05	B:44:02	C:01:02	C:05:01	DQB1:04:02	DQB1:06:03	DRB1:08:01	DRB1:13:01

**Donor name**	**Sample type**	**Cell type**								
		CD8+	CD8+ Activated	CD4+	CD4+ Activated	CD19+	CD19+ Activated	CD14+	Immatured DC	Mature DC
**D1**	Leukapheresis							x	x	x
**D2**	Apheresis filter	x		x		x		x	x	x
**D3**	Apheresis filter	x		x		x		x	x	x
**D4**	Leukapheresis	x	x	x	x	x	x	x	x	x
**D5**	Buffy coat								x	x
**D6**	Leukapheresis								x	x
**D7**	Buffy coat								x	x

**Donor name**	**Sample type**	**Experiment type**			
		Immunopeptidomics	Proteomics	Flow cytometry	Synthetic peptides loading
**D1**	Leukapheresis	x	x	x	
**D2**	Apheresis filter	x	x	x	
**D3**	Apheresis filter	x	x	x	
**D4**	Leukapheresis	x	x	x	
**D5**	Buffy coat	x			x
**D6**	Leukapheresis	x			x
**D7**	Buffy coat	x			x
