# Supplementary material for: Biogenesis of HLA Ligand Presentation in Immune Cells Upon Activation Reveals Changes in Peptide Length Preference
Source: Front Immunol. 2020 Aug 28;11:1981. doi: 10.3389/fimmu.2020.01981 (PMC7485268; doi:10.3389/fimmu.2020.01981)
Supplement: Supplementary Table 2 — Experimental design HLA class I immunopeptidomics. For each donor's HLA class I experiments, cell types, experiment type, replicate type, amount of cell used, and mass spectrometry RAW file names are listed. [file Data_Sheet_2.PDF]

Supplementary Table 2

Donor name	Cell type	Type of replicate		Experiment type	HLA	Cell amount	RAW file name
D1	CD14+	BR	MSR	Immunopeptidomics	HLA-Ip	5.00E+06	20171216_QEh1_LC1_HLAIp_SA_FaMa_Leuka2_CD14_1_R1
			MSR	Immunopeptidomics	HLA-Ip		20171216_QEh1_LC1_HLAIp_SA_FaMa_Leuka2_CD14_1_R2
		BR	MSR	Immunopeptidomics	HLA-Ip	5.00E+06	20171216_QEh1_LC1_HLAIp_SA_FaMa_Leuka2_CD14_2_R1
			MSR	Immunopeptidomics	HLA-Ip		20171216_QEh1_LC1_HLAIp_SA_FaMa_Leuka2_CD14_2_R2
		BR	MSR	Immunopeptidomics	HLA-Ip	5.00E+06	20171216_QEh1_LC1_HLAIp_SA_FaMa_Leuka2_CD14_3_R1
			MSR	Immunopeptidomics	HLA-Ip		20171216_QEh1_LC1_HLAIp_SA_FaMa_Leuka2_CD14_3_R2
	Immature DC	BR	MSR	Immunopeptidomics	HLA-Ip	5.00E+06	20171216_QEh1_LC1_HLAIp_SA_FaMa_Leuka2_ImDC_1_R1
			MSR	Immunopeptidomics	HLA-Ip		20171216_QEh1_LC1_HLAIp_SA_FaMa_Leuka2_ImDC_1_R2
		BR	MSR	Immunopeptidomics	HLA-Ip	5.00E+06	20171216_QEh1_LC1_HLAIp_SA_FaMa_Leuka2_ImDC_2_R1
			MSR	Immunopeptidomics	HLA-Ip		20171216_QEh1_LC1_HLAIp_SA_FaMa_Leuka2_ImDC_2_R2
		BR	MSR	Immunopeptidomics	HLA-Ip	5.00E+06	20171216_QEh1_LC1_HLAIp_SA_FaMa_Leuka2_ImDC_3_R1
			MSR	Immunopeptidomics	HLA-Ip		20171216_QEh1_LC1_HLAIp_SA_FaMa_Leuka2_ImDC_3_R2
	Mature DC	BR	MSR	Immunopeptidomics	HLA-Ip	5.00E+06	20171216_QEh1_LC1_HLAIp_SA_FaMa_Leuka2_MDC_1_R1
			MSR	Immunopeptidomics	HLA-Ip		20171216_QEh1_LC1_HLAIp_SA_FaMa_Leuka2_MDC_1_R2
		BR	MSR	Immunopeptidomics	HLA-Ip	5.00E+06	20171216_QEh1_LC1_HLAIp_SA_FaMa_Leuka2_MDC_2_R1
			MSR	Immunopeptidomics	HLA-Ip		20171216_QEh1_LC1_HLAIp_SA_FaMa_Leuka2_MDC_2_R2
		BR	MSR	Immunopeptidomics	HLA-Ip	5.00E+06	20171216_QEh1_LC1_HLAIp_SA_FaMa_Leuka2_MDC_3_R1
			MSR	Immunopeptidomics	HLA-Ip		20171216_QEh1_LC1_HLAIp_SA_FaMa_Leuka2_MDC_3_R2
D2	CD4+	BR	MSR	Immunopeptidomics	HLA-Ip	1.00E+07	20180424_QEh1_LC1_FaMa_HLAIp_D2_CD4_01_R1
		BR	MSR	Immunopeptidomics	HLA-Ip	1.00E+07	20180424_QEh1_LC1_FaMa_HLAIp_D2_CD4_02_R1
			MSR	Immunopeptidomics	HLA-Ip		20180424_QEh1_LC1_FaMa_HLAIp_D2_CD4_02_R2
		BR	MSR	Immunopeptidomics	HLA-Ip	1.00E+07	20180424_QEh1_LC1_FaMa_HLAIp_D2_CD4_03_R1
			MSR	Immunopeptidomics	HLA-Ip		20180424_QEh1_LC1_FaMa_HLAIp_D2_CD4_03_R2
	CD8+	BR	MSR	Immunopeptidomics	HLA-Ip	1.00E+07	20180424_QEh1_LC1_FaMa_HLAIp_D2_CD8_01_R1
			MSR	Immunopeptidomics	HLA-Ip		20180424_QEh1_LC1_FaMa_HLAIp_D2_CD8_01_R2
		BR	MSR	Immunopeptidomics	HLA-Ip	1.00E+07	20180424_QEh1_LC1_FaMa_HLAIp_D2_CD8_02_R1
			MSR	Immunopeptidomics	HLA-Ip		20180424_QEh1_LC1_FaMa_HLAIp_D2_CD8_02_R2
		BR	MSR	Immunopeptidomics	HLA-Ip	1.00E+07	20180424_QEh1_LC1_FaMa_HLAIp_D2_CD8_03_R1
	CD14+	BR	MSR	Immunopeptidomics	HLA-Ip	1.00E+07	20180424_QEh1_LC1_FaMa_HLAIp_D2_CD14_01_R1
			MSR	Immunopeptidomics	HLA-Ip		20180424_QEh1_LC1_FaMa_HLAIp_D2_CD14_01_R2
	CD19+	BR	MSR	Immunopeptidomics	HLA-Ip	1.00E+07	20180424_QEh1_LC1_FaMa_HLAIp_D2_CD19_01_R1
			MSR	Immunopeptidomics	HLA-Ip		20180424_QEh1_LC1_FaMa_HLAIp_D2_CD19_01_R2
	Immature DC	BR	MSR	Immunopeptidomics	HLA-Ip	1.00E+07	20180424_QEh1_LC1_FaMa_HLAIp_D2_ImmDc_01_R1
			MSR	Immunopeptidomics	HLA-Ip		20180424_QEh1_LC1_FaMa_HLAIp_D2_ImmDc_01_R2
	Mature DC	BR	MSR	Immunopeptidomics	HLA-Ip	1.00E+07	20180424_QEh1_LC1_FaMa_HLAIp_D2_MaDc_01_R1
			MSR	Immunopeptidomics	HLA-Ip		20180424_QEh1_LC1_FaMa_HLAIp_D2_MaDc_01_R2
D3	CD4+	BR	MSR	Immunopeptidomics	HLA-Ip	1.70E+07	20180428_QEh1_LC1_FaMa_HLAIp_D3_CD4_01_R1
			MSR	Immunopeptidomics	HLA-Ip		20180428_QEh1_LC1_FaMa_HLAIp_D3_CD4_01_R2
		BR	MSR	Immunopeptidomics	HLA-Ip	1.70E+07	20180428_QEh1_LC1_FaMa_HLAIp_D3_CD4_02_R1
			MSR	Immunopeptidomics	HLA-Ip		20180428_QEh1_LC1_FaMa_HLAIp_D3_CD4_02_R2
		BR	MSR	Immunopeptidomics	HLA-Ip	1.70E+07	20180428_QEh1_LC1_FaMa_HLAIp_D3_CD4_03_R1
			MSR	Immunopeptidomics	HLA-Ip		20180428_QEh1_LC1_FaMa_HLAIp_D3_CD4_03_R2
	CD8+	BR	MSR	Immunopeptidomics	HLA-Ip	1.70E+07	20180428_QEh1_LC1_FaMa_HLAIp_D3_CD8_01_R1
			MSR	Immunopeptidomics	HLA-Ip		20180428_QEh1_LC1_FaMa_HLAIp_D3_CD8_01_R2
		BR	MSR	Immunopeptidomics	HLA-Ip	1.70E+07	20180428_QEh1_LC1_FaMa_HLAIp_D3_CD8_02_R1
			MSR	Immunopeptidomics	HLA-Ip		20180428_QEh1_LC1_FaMa_HLAIp_D3_CD8_02_R2
	CD14+	BR	MSR	Immunopeptidomics	HLA-Ip	1.70E+07	20180428_QEh1_LC1_FaMa_HLAIp_D3_CD14_01_R1
			MSR	Immunopeptidomics	HLA-Ip		20180428_QEh1_LC1_FaMa_HLAIp_D3_CD14_01_R2
	CD19+	BR	MSR	Immunopeptidomics	HLA-Ip	1.70E+07	20180428_QEh1_LC1_FaMa_HLAIp_D3_CD19_01_R1
			MSR	Immunopeptidomics	HLA-Ip		20180428_QEh1_LC1_FaMa_HLAIp_D3_CD19_01_R2
		BR	MSR	Immunopeptidomics	HLA-Ip	1.70E+07	20180428_QEh1_LC1_FaMa_HLAIp_D3_CD19_02_R1
			MSR	Immunopeptidomics	HLA-Ip		20180428_QEh1_LC1_FaMa_HLAIp_D3_CD19_02_R2
	Immature DC	BR	MSR	Immunopeptidomics	HLA-Ip	1.70E+07	20180428_QEh1_LC1_FaMa_HLAIp_D3_ImmDc_01_R1
			MSR	Immunopeptidomics	HLA-Ip		20180428_QEh1_LC1_FaMa_HLAIp_D3_ImmDc_01_R2
	Mature DC	BR	MSR	Immunopeptidomics	HLA-Ip	1.70E+07	20180428_QEh1_LC1_FaMa_HLAIp_D3_MaDc_01_R1
			MSR	Immunopeptidomics	HLA-Ip		20180428_QEh1_LC1_FaMa_HLAIp_D3_MaDc_01_R2
D4	CD4+	BR	MSR	Immunopeptidomics	HLA-Ip	1.20E+07	20180814_QEh1_LC1_SA_FaMa_HLAIp_CD4_1_R1
			MSR	Immunopeptidomics	HLA-Ip		20180814_QEh1_LC1_SA_FaMa_HLAIp_CD4_1_R2
		BR	MSR	Immunopeptidomics	HLA-Ip	1.20E+07	20180814_QEh1_LC1_SA_FaMa_HLAIp_CD4_2_R1
			MSR	Immunopeptidomics	HLA-Ip		20180814_QEh1_LC1_SA_FaMa_HLAIp_CD4_2_R2
		BR	MSR	Immunopeptidomics	HLA-Ip	1.20E+07	20180814_QEh1_LC1_SA_FaMa_HLAIp_CD4_3_R1
			MSR	Immunopeptidomics	HLA-Ip		20180814_QEh1_LC1_SA_FaMa_HLAIp_CD4_3_R2
	CD4+ Activated	BR	MSR	Immunopeptidomics	HLA-Ip	1.20E+07	20180814_QEh1_LC1_SA_FaMa_HLAIp_CD4_Act_1_R1
			MSR	Immunopeptidomics	HLA-Ip		20180814_QEh1_LC1_SA_FaMa_HLAIp_CD4_Act_1_R2
		BR	MSR	Immunopeptidomics	HLA-Ip	1.20E+07	20180814_QEh1_LC1_SA_FaMa_HLAIp_CD4_Act_2_R1
			MSR	Immunopeptidomics	HLA-Ip		20180814_QEh1_LC1_SA_FaMa_HLAIp_CD4_Act_2_R2
		BR	MSR	Immunopeptidomics	HLA-Ip	1.20E+07	20180814_QEh1_LC1_SA_FaMa_HLAIp_CD4_Act_3_R1
			MSR	Immunopeptidomics	HLA-Ip		20180814_QEh1_LC1_SA_FaMa_HLAIp_CD4_Act_3_R2
	CD8+	BR	MSR	Immunopeptidomics	HLA-Ip	1.20E+07	20180814_QEh1_LC1_SA_FaMa_HLAIp_CD8_1_R1
			MSR	Immunopeptidomics	HLA-Ip		20180814_QEh1_LC1_SA_FaMa_HLAIp_CD8_1_R2
		BR	MSR	Immunopeptidomics	HLA-Ip	1.20E+07	20180814_QEh1_LC1_SA_FaMa_HLAIp_CD8_2_R1
			MSR	Immunopeptidomics	HLA-Ip		20180814_QEh1_LC1_SA_FaMa_HLAIp_CD8_2_R2
		BR	MSR	Immunopeptidomics	HLA-Ip	1.20E+07	20180814_QEh1_LC1_SA_FaMa_HLAIp_CD8_3_R1
			MSR	Immunopeptidomics	HLA-Ip		20180814_QEh1_LC1_SA_FaMa_HLAIp_CD8_3_R2
	CD8+ Activated	BR	MSR	Immunopeptidomics	HLA-Ip	1.20E+07	20180814_QEh1_LC1_SA_FaMa_HLAIp_CD8_Act_1_R1
			MSR	Immunopeptidomics	HLA-Ip		20180814_QEh1_LC1_SA_FaMa_HLAIp_CD8_Act_1_R2
		BR	MSR	Immunopeptidomics	HLA-Ip	1.20E+07	20180814_QEh1_LC1_SA_FaMa_HLAIp_CD8_Act_2_R1
			MSR	Immunopeptidomics	HLA-Ip		20180814_QEh1_LC1_SA_FaMa_HLAIp_CD8_Act_2_R2
		BR	MSR	Immunopeptidomics	HLA-Ip	1.20E+07	20180814_QEh1_LC1_SA_FaMa_HLAIp_CD8_Act_3_R1
			MSR	Immunopeptidomics	HLA-Ip		20180814_QEh1_LC1_SA_FaMa_HLAIp_CD8_Act_3_R2
	CD14+	BR	MSR	Immunopeptidomics	HLA-Ip	1.20E+07	20180814_QEh1_LC1_SA_FaMa_HLAIp_CD14_1_R1
			MSR	Immunopeptidomics	HLA-Ip		20180814_QEh1_LC1_SA_FaMa_HLAIp_CD14_1_R2
		BR	MSR	Immunopeptidomics	HLA-Ip	1.20E+07	20180814_QEh1_LC1_SA_FaMa_HLAIp_CD14_2_R1
			MSR	Immunopeptidomics	HLA-Ip		20180814_QEh1_LC1_SA_FaMa_HLAIp_CD14_2_R2
		BR	MSR	Immunopeptidomics	HLA-Ip	1.20E+07	20180814_QEh1_LC1_SA_FaMa_HLAIp_CD14_3_R1
			MSR	Immunopeptidomics	HLA-Ip		20180814_QEh1_LC1_SA_FaMa_HLAIp_CD14_3_R2
	CD19+	BR	MSR	Immunopeptidomics	HLA-Ip	1.20E+07	20180814_QEh1_LC1_SA_FaMa_HLAIp_CD19_1_R1
			MSR	Immunopeptidomics	HLA-Ip		20180814_QEh1_LC1_SA_FaMa_HLAIp_CD19_1_R2
		BR	MSR	Immunopeptidomics	HLA-Ip	1.20E+07	20180814_QEh1_LC1_SA_FaMa_HLAIp_CD19_2_R1
			MSR	Immunopeptidomics	HLA-Ip		20180814_QEh1_LC1_SA_FaMa_HLAIp_CD19_2_R2
		BR	MSR	Immunopeptidomics	HLA-Ip	1.20E+07	20180814_QEh1_LC1_SA_FaMa_HLAIp_CD19_3_R1
			MSR	Immunopeptidomics	HLA-Ip		20180814_QEh1_LC1_SA_FaMa_HLAIp_CD19_3_R2
	CD19+ Activated	BR	MSR	Immunopeptidomics	HLA-Ip	1.20E+07	20180814_QEh1_LC1_SA_FaMa_HLAIp_CD19_Act_1_R1
			MSR	Immunopeptidomics	HLA-Ip		20180814_QEh1_LC1_SA_FaMa_HLAIp_CD19_Act_1_R2
	Immature DC	BR	MSR	Immunopeptidomics	HLA-Ip	1.20E+07	20180814_QEh1_LC1_SA_FaMa_HLAIp_ImmDC_1_R1
			MSR	Immunopeptidomics	HLA-Ip		20180814_QEh1_LC1_SA_FaMa_HLAIp_ImmDC_1_R2

	Mature DC	BR	MSR	Immunopeptidomics	HLA-Ip	1.20E+07	20180814_QEh1_LC1_SA_FaMa_HLAIp_MaDC_1_R1
			MSR	Immunopeptidomics	HLA-Ip		20180814_QEh1_LC1_SA_FaMa_HLAIp_MaDC_1_R2

BR: Separate cells pellets or cultured in different flasks

MSR: measured twice are MS replicates

HLA-Ip: HLA class I peptides
