# Supplementary material for: Biogenesis of HLA Ligand Presentation in Immune Cells Upon Activation Reveals Changes in Peptide Length Preference
Source: Front Immunol. 2020 Aug 28;11:1981. doi: 10.3389/fimmu.2020.01981 (PMC7485268; doi:10.3389/fimmu.2020.01981)
Supplement: Supplementary Table 3 — Experimental design HLA class II immunopeptidomics. For each donor's HLA class II experiments, cell types, experiment type, replicate type, amount of cell used, and mass spectrometry RAW file names are listed. [file Data_Sheet_3.PDF]

Supplementary Table 3

Donor name	Cell type	Type of replicate		Experiment type	HLA	Cell amount	RAW file name
D1	CD14+	BR	MSR	Immunopeptidomics	HLA-IIp	5.00E+06	20171216_QEh1_LC1_HLAIIp_SA_FaMa_Leuka2_CD14_1_R1
			MSR	Immunopeptidomics	HLA-IIp		20171216_QEh1_LC1_HLAIIp_SA_FaMa_Leuka2_CD14_1_R2
		BR	MSR	Immunopeptidomics	HLA-IIp	5.00E+06	20171216_QEh1_LC1_HLAIIp_SA_FaMa_Leuka2_CD14_2_R1
			MSR	Immunopeptidomics	HLA-IIp		20171216_QEh1_LC1_HLAIIp_SA_FaMa_Leuka2_CD14_2_R2
		BR	MSR	Immunopeptidomics	HLA-IIp	5.00E+06	20171216_QEh1_LC1_HLAIIp_SA_FaMa_Leuka2_CD14_3_R1
			MSR	Immunopeptidomics	HLA-IIp		20171216_QEh1_LC1_HLAIIp_SA_FaMa_Leuka2_CD14_3_R2
	Immature DC	BR	MSR	Immunopeptidomics	HLA-IIp	5.00E+06	20171216_QEh1_LC1_HLAIIp_SA_FaMa_Leuka2_ImDC_1_R1
			MSR	Immunopeptidomics	HLA-IIp		20171216_QEh1_LC1_HLAIIp_SA_FaMa_Leuka2_ImDC_1_R2
		BR	MSR	Immunopeptidomics	HLA-IIp	5.00E+06	20171216_QEh1_LC1_HLAIIp_SA_FaMa_Leuka2_ImDC_2_R1
			MSR	Immunopeptidomics	HLA-IIp		20171216_QEh1_LC1_HLAIIp_SA_FaMa_Leuka2_ImDC_2_R2
		BR	MSR	Immunopeptidomics	HLA-IIp	5.00E+06	20171216_QEh1_LC1_HLAIIp_SA_FaMa_Leuka2_ImDC_3_R1
			MSR	Immunopeptidomics	HLA-IIp		20171216_QEh1_LC1_HLAIIp_SA_FaMa_Leuka2_ImDC_3_R2
	Mature DC	BR	MSR	Immunopeptidomics	HLA-IIp	5.00E+06	20171216_QEh1_LC1_HLAIIp_SA_FaMa_Leuka2_MDC_1_R1
			MSR	Immunopeptidomics	HLA-IIp		20171216_QEh1_LC1_HLAIIp_SA_FaMa_Leuka2_MDC_1_R2
		BR	MSR	Immunopeptidomics	HLA-IIp	5.00E+06	20171216_QEh1_LC1_HLAIIp_SA_FaMa_Leuka2_MDC_2_R1
			MSR	Immunopeptidomics	HLA-IIp		20171216_QEh1_LC1_HLAIIp_SA_FaMa_Leuka2_MDC_2_R2
		BR	MSR	Immunopeptidomics	HLA-IIp	5.00E+06	20171216_QEh1_LC1_HLAIIp_SA_FaMa_Leuka2_MDC_3_R1
			MSR	Immunopeptidomics	HLA-IIp		20171216_QEh1_LC1_HLAIIp_SA_FaMa_Leuka2_MDC_3_R2
D2	CD4+	BR	MSR	Immunopeptidomics	HLA-IIp	1.00E+07	20180427_QEh1_LC1_FaMa_HLAIIp_D2_CD4_02_R1
			MSR	Immunopeptidomics	HLA-IIp		20180427_QEh1_LC1_FaMa_HLAIIp_D2_CD4_02_R2
		BR	MSR	Immunopeptidomics	HLA-IIp	1.00E+07	20180427_QEh1_LC1_FaMa_HLAIIp_D2_CD4_03_R1
			MSR	Immunopeptidomics	HLA-IIp		20180427_QEh1_LC1_FaMa_HLAIIp_D2_CD4_03_R2
	CD8+	BR	MSR	Immunopeptidomics	HLA-IIp	1.00E+07	20180427_QEh1_LC1_FaMa_HLAIIp_D2_CD8_01_R1
			MSR	Immunopeptidomics	HLA-IIp		20180427_QEh1_LC1_FaMa_HLAIIp_D2_CD8_01_R2
		BR	MSR	Immunopeptidomics	HLA-IIp	1.00E+07	20180427_QEh1_LC1_FaMa_HLAIIp_D2_CD8_02_R1
			MSR	Immunopeptidomics	HLA-IIp		20180427_QEh1_LC1_FaMa_HLAIIp_D2_CD8_02_R2
		BR	MSR	Immunopeptidomics	HLA-IIp	1.00E+07	20180427_QEh1_LC1_FaMa_HLAIIp_D2_CD8_03_R1
			MSR	Immunopeptidomics	HLA-IIp		20180427_QEh1_LC1_FaMa_HLAIIp_D2_CD8_03_R2
	CD14+	BR	MSR	Immunopeptidomics	HLA-IIp	1.00E+07	20180426_QEh1_LC1_FaMa_HLAIIp_D2_CD14_01_R1
			MSR	Immunopeptidomics	HLA-IIp		20180426_QEh1_LC1_FaMa_HLAIIp_D2_CD14_01_R2
	CD19+	BR	MSR	Immunopeptidomics	HLA-IIp	1.00E+07	20180426_QEh1_LC1_FaMa_HLAIIp_D2_CD19_01_R1
			MSR	Immunopeptidomics	HLA-IIp		20180426_QEh1_LC1_FaMa_HLAIIp_D2_CD19_01_R2
	Immature DC	BR	MSR	Immunopeptidomics	HLA-IIp	1.70E+07	20180424_QEh1_LC1_FaMa_HLAIIp_D2_ImmDc_01_R1
			MSR	Immunopeptidomics	HLA-IIp		20180424_QEh1_LC1_FaMa_HLAIIp_D2_ImmDc_01_R2_180427070717
	Mature DC	BR	MSR	Immunopeptidomics	HLA-IIp	1.70E+07	20180426_QEh1_LC1_FaMa_HLAIIp_D2_MaDc_01_R1
			MSR	Immunopeptidomics	HLA-IIp		20180426_QEh1_LC1_FaMa_HLAIIp_D2_MaDc_01_R2
D3	CD4+	BR	MSR	Immunopeptidomics	HLA-IIp	1.70E+07	20180428_QEh1_LC1_FaMa_HLAIIp_D3_CD4_01_R1
			MSR	Immunopeptidomics	HLA-IIp		20180428_QEh1_LC1_FaMa_HLAIIp_D3_CD4_01_R2
		BR	MSR	Immunopeptidomics	HLA-IIp	1.70E+07	20180428_QEh1_LC1_FaMa_HLAIIp_D3_CD4_02_R1
			MSR	Immunopeptidomics	HLA-IIp		20180428_QEh1_LC1_FaMa_HLAIIp_D3_CD4_02_R2
		BR	MSR	Immunopeptidomics	HLA-IIp	1.70E+07	20180428_QEh1_LC1_FaMa_HLAIIp_D3_CD4_03_R1
			MSR	Immunopeptidomics	HLA-IIp		20180428_QEh1_LC1_FaMa_HLAIIp_D3_CD4_03_R2
	CD8+	BR	MSR	Immunopeptidomics	HLA-IIp	1.70E+07	20180428_QEh1_LC1_FaMa_HLAIIp_D3_CD8_01_R1
			MSR	Immunopeptidomics	HLA-IIp		20180428_QEh1_LC1_FaMa_HLAIIp_D3_CD8_01_R2
		BR	MSR	Immunopeptidomics	HLA-IIp	1.70E+07	20180428_QEh1_LC1_FaMa_HLAIIp_D3_CD8_02_R1
			MSR	Immunopeptidomics	HLA-IIp		20180428_QEh1_LC1_FaMa_HLAIIp_D3_CD8_02_R2
	CD14+	BR	MSR	Immunopeptidomics	HLA-IIp	1.70E+07	20180428_QEh1_LC1_FaMa_HLAIIp_D3_CD14_01_R1
			MSR	Immunopeptidomics	HLA-IIp		20180428_QEh1_LC1_FaMa_HLAIIp_D3_CD14_01_R2_180501121229
	CD19+	BR	MSR	Immunopeptidomics	HLA-IIp	1.70E+07	20180428_QEh1_LC1_FaMa_HLAIIp_D3_CD19_01_R1
			MSR	Immunopeptidomics	HLA-IIp		20180428_QEh1_LC1_FaMa_HLAIIp_D3_CD19_01_R2
		BR	MSR	Immunopeptidomics	HLA-IIp	1.70E+07	20180428_QEh1_LC1_FaMa_HLAIIp_D3_CD19_02_R1
			MSR	Immunopeptidomics	HLA-IIp		20180428_QEh1_LC1_FaMa_HLAIIp_D3_CD19_02_R2
	Immature DC	BR	MSR	Immunopeptidomics	HLA-IIp	1.20E+07	20180428_QEh1_LC1_FaMa_HLAIIp_D3_ImmDc_01_R1
			MSR	Immunopeptidomics	HLA-IIp		20180428_QEh1_LC1_FaMa_HLAIIp_D3_ImmDc_01_R2
	Mature DC	BR	MSR	Immunopeptidomics	HLA-IIp	1.20E+07	20180428_QEh1_LC1_FaMa_HLAIIp_D3_MaDc_01_R1
			MSR	Immunopeptidomics	HLA-IIp		20180428_QEh1_LC1_FaMa_HLAIIp_D3_MaDc_01_R2
D4	CD4+	BR	MSR	Immunopeptidomics	HLA-IIp	1.20E+07	20180814_QEh1_LC1_SA_FaMa_HLAIIp_CD4_1_R1
			MSR	Immunopeptidomics	HLA-IIp		20180814_QEh1_LC1_SA_FaMa_HLAIIp_CD4_1_R2
		BR	MSR	Immunopeptidomics	HLA-IIp	1.20E+07	20180814_QEh1_LC1_SA_FaMa_HLAIIp_CD4_2_R1
			MSR	Immunopeptidomics	HLA-IIp		20180814_QEh1_LC1_SA_FaMa_HLAIIp_CD4_2_R2
		BR	MSR	Immunopeptidomics	HLA-IIp	1.20E+07	20180814_QEh1_LC1_SA_FaMa_HLAIIp_CD4_3_R1
			MSR	Immunopeptidomics	HLA-IIp		20180814_QEh1_LC1_SA_FaMa_HLAIIp_CD4_3_R2
	CD4+ Activated	BR	MSR	Immunopeptidomics	HLA-IIp	1.20E+07	20180814_QEh1_LC1_SA_FaMa_HLAIIp_CD4_Act_1_R1
			MSR	Immunopeptidomics	HLA-IIp		20180814_QEh1_LC1_SA_FaMa_HLAIIp_CD4_Act_1_R2
		BR	MSR	Immunopeptidomics	HLA-IIp	1.20E+07	20180814_QEh1_LC1_SA_FaMa_HLAIIp_CD4_Act_2_R1
			MSR	Immunopeptidomics	HLA-IIp		20180814_QEh1_LC1_SA_FaMa_HLAIIp_CD4_Act_2_R2
		BR	MSR	Immunopeptidomics	HLA-IIp	1.20E+07	20180814_QEh1_LC1_SA_FaMa_HLAIIp_CD4_Act_3_R1
			MSR	Immunopeptidomics	HLA-IIp		20180814_QEh1_LC1_SA_FaMa_HLAIIp_CD4_Act_3_R2
	CD8+	BR	MSR	Immunopeptidomics	HLA-IIp	1.20E+07	20180814_QEh1_LC1_SA_FaMa_HLAIIp_CD8_1_R1
			MSR	Immunopeptidomics	HLA-IIp		20180814_QEh1_LC1_SA_FaMa_HLAIIp_CD8_1_R2
		BR	MSR	Immunopeptidomics	HLA-IIp	1.20E+07	20180814_QEh1_LC1_SA_FaMa_HLAIIp_CD8_2_R1
			MSR	Immunopeptidomics	HLA-IIp		20180814_QEh1_LC1_SA_FaMa_HLAIIp_CD8_2_R2
		BR	MSR	Immunopeptidomics	HLA-IIp	1.20E+07	20180814_QEh1_LC1_SA_FaMa_HLAIIp_CD8_3_R1
			MSR	Immunopeptidomics	HLA-IIp		20180814_QEh1_LC1_SA_FaMa_HLAIIp_CD8_3_R2
	CD8+ Activated	BR	MSR	Immunopeptidomics	HLA-IIp	1.20E+07	20180814_QEh1_LC1_SA_FaMa_HLAIIp_CD8_Act_1_R1
			MSR	Immunopeptidomics	HLA-IIp		20180814_QEh1_LC1_SA_FaMa_HLAIIp_CD8_Act_1_R2
		BR	MSR	Immunopeptidomics	HLA-IIp	1.20E+07	20180814_QEh1_LC1_SA_FaMa_HLAIIp_CD8_Act_2_R1
		BR	MSR	Immunopeptidomics	HLA-IIp	1.20E+07	20180814_QEh1_LC1_SA_FaMa_HLAIIp_CD8_Act_3_R1
			MSR	Immunopeptidomics	HLA-IIp		20180814_QEh1_LC1_SA_FaMa_HLAIIp_CD8_Act_3_R2
	CD14+	BR	MSR	Immunopeptidomics	HLA-IIp	1.20E+07	20180814_QEh1_LC1_SA_FaMa_HLAIIp_CD14_1_R1
			MSR	Immunopeptidomics	HLA-IIp		20180814_QEh1_LC1_SA_FaMa_HLAIIp_CD14_1_R2
		BR	MSR	Immunopeptidomics	HLA-IIp	1.20E+07	20180814_QEh1_LC1_SA_FaMa_HLAIIp_CD14_2_R1
			MSR	Immunopeptidomics	HLA-IIp		20180814_QEh1_LC1_SA_FaMa_HLAIIp_CD14_2_R2
		BR	MSR	Immunopeptidomics	HLA-IIp	1.20E+07	20180814_QEh1_LC1_SA_FaMa_HLAIIp_CD14_3_R1
			MSR	Immunopeptidomics	HLA-IIp		20180814_QEh1_LC1_SA_FaMa_HLAIIp_CD14_3_R2
	CD19+	BR	MSR	Immunopeptidomics	HLA-IIp	1.20E+07	20180814_QEh1_LC1_SA_FaMa_HLAIIp_CD19_1_R1
		BR	MSR	Immunopeptidomics	HLA-IIp	1.20E+07	20180814_QEh1_LC1_SA_FaMa_HLAIIp_CD19_2_R1
			MSR	Immunopeptidomics	HLA-IIp		20180814_QEh1_LC1_SA_FaMa_HLAIIp_CD19_2_R2
		BR	MSR	Immunopeptidomics	HLA-IIp	1.20E+07	20180814_QEh1_LC1_SA_FaMa_HLAIIp_CD19_3_R1
			MSR	Immunopeptidomics	HLA-IIp		20180814_QEh1_LC1_SA_FaMa_HLAIIp_CD19_3_R2
	CD19+ Activated	BR	MSR	Immunopeptidomics	HLA-IIp	1.20E+07	20180814_QEh1_LC1_SA_FaMa_HLAIIp_CD19_Act_1_R1
			MSR	Immunopeptidomics	HLA-IIp		20180814_QEh1_LC1_SA_FaMa_HLAIIp_CD19_Act_1_R2
	Immature DC	BR	MSR	Immunopeptidomics	HLA-IIp	1.20E+07	20180814_QEh1_LC1_SA_FaMa_HLAIIp_ImmDC_1_R1
			MSR	Immunopeptidomics	HLA-IIp		20180814_QEh1_LC1_SA_FaMa_HLAIIp_ImmDC_1_R2
	Mature DC	BR	MSR	Immunopeptidomics	HLA-IIp	1.20E+07	20180814_QEh1_LC1_SA_FaMa_HLAIIp_MaDC_1_R1
			MSR	Immunopeptidomics	HLA-IIp		20180814_QEh1_LC1_SA_FaMa_HLAIIp_MaDC_1_R2
D5	Immature DC	BR	MSR	Immunopeptidomics	HLA-IIp	7.00E+06	20181029_QEh1_LC1_SA_FaMa_HLAIIp_ImmDC_10ug_D4_1_R1
			MSR	Immunopeptidomics	HLA-IIp		20181029_QEh1_LC1_SA_FaMa_HLAIIp_ImmDC_10ug_D4_2_R1
	Mature DC	BR	MSR	Immunopeptidomics	HLA-IIp	7.00E+06	20181029_QEh1_LC1_SA_FaMa_HLAIIp_MaDC_10ug_D4_1_R1
			MSR	Immunopeptidomics	HLA-IIp		20181029_QEh1_LC1_SA_FaMa_HLAIIp_MaDC_10ug_D4_2_R1

BR: Separate cells pellets or cultured in different flasks

MSR: measured twice are MS replicates
HLA-IIp: HLA class II peptides
