# Supplementary material for: Biogenesis of HLA Ligand Presentation in Immune Cells Upon Activation Reveals Changes in Peptide Length Preference
Source: Front Immunol. 2020 Aug 28;11:1981. doi: 10.3389/fimmu.2020.01981 (PMC7485268; doi:10.3389/fimmu.2020.01981)
Supplement: Supplementary Table 4 — Experimental design proteomics. For each donor's proteomics experiments, cell types, experiment type, replicate type, amount of cell used, and mass spectrometry RAW file names are listed. [file Data_Sheet_4.PDF]

Supplementary Table 4

Donor name	Cell type	Type of replicate		Experiment type	Cell amount	RAW file name
D1	CD14+	BR	MSR	Proteomics	1.00E+06	20171222_QE_HFX_LC2_Prot_SA_FaMa_Leuka2_CD14_1_R1
			MSR	Proteomics		20171222_QE_HFX_LC2_Prot_SA_FaMa_Leuka2_CD14_1_R2
		BR	MSR	Proteomics	1.00E+06	20171222_QE_HFX_LC2_Prot_SA_FaMa_Leuka2_CD14_2_R1
			MSR	Proteomics		20171222_QE_HFX_LC2_Prot_SA_FaMa_Leuka2_CD14_2_R2
		BR	MSR	Proteomics	1.00E+06	20171222_QE_HFX_LC2_Prot_SA_FaMa_Leuka2_ImmDC_1_R1
			MSR	Proteomics		20171222_QE_HFX_LC2_Prot_SA_FaMa_Leuka2_ImmDC_1_R2
	Immature DC	BR	MSR	Proteomics	1.00E+06	20171222_QE_HFX_LC2_Prot_SA_FaMa_Leuka2_ImmDC_2_R1
		BR	MSR	Proteomics	1.00E+06	20171222_QE_HFX_LC2_Prot_SA_FaMa_Leuka2_ImmDC_3_R1
			MSR	Proteomics		20171222_QE_HFX_LC2_Prot_SA_FaMa_Leuka2_ImmDC_3_R2
	Mature DC	BR	MSR	Proteomics	1.00E+06	20171222_QE_HFX_LC2_Prot_SA_FaMa_Leuka2_MDC_1_R1
			MSR	Proteomics		20171222_QE_HFX_LC2_Prot_SA_FaMa_Leuka2_MDC_1_R2
		BR	MSR	Proteomics	1.00E+06	20171222_QE_HFX_LC2_Prot_SA_FaMa_Leuka2_MDC_2_R1
			MSR	Proteomics		20171222_QE_HFX_LC2_Prot_SA_FaMa_Leuka2_MDC_2_R2
		BR	MSR	Proteomics	1.00E+06	20171222_QE_HFX_LC2_Prot_SA_FaMa_Leuka2_MDC_3_R1
			MSR	Proteomics		20171222_QE_HFX_LC2_Prot_SA_FaMa_Leuka2_MDC_3_R2
D2	CD14+	BR	MSR	Proteomics	1.00E+06	20180622_QEh1_LC1_SA_FaMa_Prot_D2_CD14_1_R01
			MSR	Proteomics		20180622_QEh1_LC1_SA_FaMa_Prot_D2_CD14_1_R02
	Immature DC	BR	MSR	Proteomics	1.00E+06	20180622_QEh1_LC1_SA_FaMa_Prot_D2_ImmDC_1_R01
			MSR	Proteomics		20180622_QEh1_LC1_SA_FaMa_Prot_D2_ImmDC_1_R02
	Mature DC	BR	MSR	Proteomics	1.00E+06	20180509_QE_HFX_LC2_Prot_FaMa_SA_MaDC_D2_1_R1
			MSR	Proteomics		20180509_QE_HFX_LC2_Prot_FaMa_SA_MaDC_D2_1_R2
D3	CD14+	BR	MSR	Proteomics	1.00E+06	20180808_QEh1_LC1_SA_FaMa_Prot_CD14_D3_1_R01
			MSR	Proteomics		20180808_QEh1_LC1_SA_FaMa_Prot_CD14_D3_1_R02
	Immature DC	BR	MSR	Proteomics	1.00E+06	20180808_QEh1_LC1_SA_FaMa_Prot_ImmDC_D3_1_R01
			MSR	Proteomics		20180808_QEh1_LC1_SA_FaMa_Prot_ImmDC_D3_1_R02
	Mature DC	BR	MSR	Proteomics	1.00E+06	20180808_QEh1_LC1_SA_FaMa_Prot_maDC_D3_1_R01
			MSR	Proteomics		20180808_QEh1_LC1_SA_FaMa_Prot_maDC_D3_1_R02
D4	CD4+ Activated	BR	MSR	Proteomics	1.00E+06	20180710_QEh1_LC1_SA_FaMa_CD4_Act_Leuka3_1_R1
			MSR	Proteomics		20180710_QEh1_LC1_SA_FaMa_CD4_Act_Leuka3_1_R2
	CD4+	BR	MSR	Proteomics	1.00E+06	20180710_QEh1_LC1_SA_FaMa_CD4_Leuka3_2_R1
			MSR	Proteomics		20180710_QEh1_LC1_SA_FaMa_CD4_Leuka3_2_R2
	CD8+ Activated	BR	MSR	Proteomics	1.00E+06	20180710_QEh1_LC1_SA_FaMa_CD8_Act_Leuka3_1_R1
			MSR	Proteomics		20180710_QEh1_LC1_SA_FaMa_CD8_Act_Leuka3_1_R2
	CD8+	BR	MSR	Proteomics	1.00E+06	20180710_QEh1_LC1_SA_FaMa_CD8_Leuka3_2_R1
			MSR	Proteomics		20180710_QEh1_LC1_SA_FaMa_CD8_Leuka3_2_R2
	CD14+	BR	MSR	Proteomics	1.00E+06	20180710_QEh1_LC1_SA_FaMa_CD14_Leuka3_1_R1
			MSR	Proteomics		20180710_QEh1_LC1_SA_FaMa_CD14_Leuka3_1_R2
	CD19+ Activated	BR	MSR	Proteomics	1.00E+06	20180710_QEh1_LC1_SA_FaMa_CD19_Act_Leuka3_1_R1
			MSR	Proteomics		20180710_QEh1_LC1_SA_FaMa_CD19_Act_Leuka3_1_R2
	CD19+	BR	MSR	Proteomics	1.00E+06	20180710_QEh1_LC1_SA_FaMa_CD19_Leuka3_2_R1
			MSR	Proteomics		20180710_QEh1_LC1_SA_FaMa_CD19_Leuka3_2_R2
	Immature DC	BR	MSR	Proteomics	1.00E+06	20180710_QEh1_LC1_SA_FaMa_ImmDC_Leuka3_1_R1
			MSR	Proteomics		20180710_QEh1_LC1_SA_FaMa_ImmDC_Leuka3_1_R2
	Mature DC	BR	MSR	Proteomics	1.00E+06	20180710_QEh1_LC1_SA_FaMa_MaDC_Leuka3_1_R1
			MSR	Proteomics		20180710_QEh1_LC1_SA_FaMa_MaDC_Leuka3_1_R2

BR: Separate cells pellets or cultured in different flasks

MSR: measured twice are MS replicates
