# Supplementary material for: Biogenesis of HLA Ligand Presentation in Immune Cells Upon Activation Reveals Changes in Peptide Length Preference
Source: Front Immunol. 2020 Aug 28;11:1981. doi: 10.3389/fimmu.2020.01981 (PMC7485268; doi:10.3389/fimmu.2020.01981)
Supplement: Supplementary Table 8 — GO enrichment of molecular functions upon DC differentiation. GO enrichment of molecular functions was calculated by String (version 10.5). For this analysis, from each donor were used proteins found to be significantly upregulated (FDR = 1%, S0 = 3) upon monocyte differentiation to immature dendritic cells in proteomics analysis. The top 20 enriched functions are reported. [file Data_Sheet_8.PDF]

Supplementary Table 8

GO ID	GO name	observed protein count	P-Value
**Top 20 Donor D1 enriched for upregulated proteins in Immature dendritic cells**			
GO.003303	macromolecule localization	158	3.09E-21
GO.001503	protein transport	116	3.10E-21
GO.001619	vesicle-mediated transport	110	1.10E-20
GO.004518	establishment of protein localization	119	1.10E-20
GO.000810	protein localization	138	1.78E-20
GO.007170	organic substance transport	147	2.42E-20
GO.004690	intracellular transport	109	4.67E-16
GO.005164	cellular localization	146	1.69E-15
GO.190258	single-organism intracellular transport	98	4.44E-15
GO.005164	establishment of localization in cell	128	6.84E-15
GO.190157	organic substance catabolic process	116	1.15E-14
GO.000905	catabolic process	127	1.82E-14
GO.000681	transport	198	3.25E-14
GO.004424	cellular catabolic process	111	3.29E-14
GO.004440	symbiosis, encompassing mutualism throu	77	4.02E-14
GO.001603	viral process	72	6.99E-14
GO.005123	establishment of localization	201	7.21E-14
GO.005117	localization	231	8.18E-14
GO.006102	membrane organization	79	3.81E-13
GO.000689	endocytosis	57	5.94E-13
**Top 20 Donor D1 Enriched for upregulated proteins in CD14+ cells**			
GO.002290	respiratory electron transport chain	19	8.69E-13
GO.004533	cellular respiration	21	9.44E-13
GO.005123	establishment of localization	82	2.40E-11
GO.190257	single-organism localization	75	2.90E-11
GO.000681	transport	78	2.84E-10
GO.004476	single-organism transport	69	1.15E-09
GO.001598	energy derivation by oxidation of organic	23	1.33E-09
GO.000695	response to stress	75	6.25E-09
GO.005117	localization	86	7.02E-09
GO.000609	generation of precursor metabolites and e	24	7.10E-09
GO.004476	single-organism cellular process	147	7.57E-09
GO.000177	cell activation	29	1.22E-08
GO.005164	cellular localization	54	4.99E-08
GO.005511	oxidation-reduction process	35	9.94E-08
GO.004508	innate immune response	33	3.34E-07
GO.000695	defense response	41	4.38E-07
GO.000683	mitochondrial transport	14	7.01E-07
GO.000920	purine ribonucleoside triphosphate metab	15	7.01E-07
GO.004469	single-organism process	146	7.01E-07
GO.005164	establishment of localization in cell	46	7.01E-07

GO ID	GO name	observed protein count	P-Value
**Top 20 Donor D4 enriched for upregulated proteins in Immature dendritic cells**			
GO.004428	small molecule metabolic process	77	1.47E-10

GO.001975	carboxylic acid metabolic process	42	2.41E-09
GO.004424	cellular catabolic process	58	2.41E-09
GO.000905	catabolic process	64	5.52E-09
GO.003303	macromolecule localization	70	5.52E-09
GO.005117	localization	112	7.62E-09
GO.190157	organic substance catabolic process	58	8.20E-09
GO.006102	membrane organization	42	1.17E-08
GO.004343	oxoacid metabolic process	43	1.44E-08
GO.004471	single-organism catabolic process	40	1.72E-07
GO.004690	intracellular transport	50	1.90E-07
GO.005164	cellular localization	67	1.90E-07
GO.004471	single-organism metabolic process	105	2.38E-07
GO.000810	protein localization	58	2.50E-07
GO.007097	protein localization to endoplasmic reticu	16	2.50E-07
GO.001619	vesicle-mediated transport	45	5.80E-07
GO.007170	organic substance transport	61	5.80E-07
GO.005511	oxidation-reduction process	41	9.60E-07
GO.000681	transport	89	1.24E-06
GO.004504	protein targeting to ER	14	1.24E-06
**Top 20 Donor D4 enriched for upregulated proteins in CD14+ cells**			
GO.004274	defense response to bacterium	13	1.74E-05
GO.004427	cellular nitrogen compound catabolic proc	17	1.74E-05
GO.000695	inflammatory response	17	5.56E-05
GO.000257	platelet degranulation	9	5.99E-05
GO.000177	cell activation	20	6.01E-05
GO.000961	response to bacterium	17	6.62E-05
GO.003465	nucleobase-containing compound catabol	15	6.62E-05
GO.000627	DNA strand elongation involved in DNA re	6	0.000189
GO.000960	response to biotic stimulus	20	0.000189
GO.000695	response to stress	49	0.000214
GO.005127	chromosome organization	22	0.000236
GO.005170	response to other organism	19	0.000281
GO.001619	vesicle-mediated transport	25	0.000316
GO.007110	DNA conformation change	11	0.000354
GO.000960	response to external stimulus	33	0.000359
GO.000237	immune system process	34	0.000398
GO.000626	DNA unwinding involved in DNA replicatio	4	0.000413
GO.000695	immune response	26	0.000827
GO.000695	defense response	27	0.000838
GO.000759	hemostasis	16	0.000921

GO ID	GO name	observed protein count	P-Value
**Top 20 Donor D2 Enriched for upregulated proteins in Immature dendritic cells**			
GO.000689	endocytosis	20	5.57E-06
GO.001619	vesicle-mediated transport	29	7.26E-06
GO.006500	regulation of biological quality	48	2.42E-05
GO.000689	receptor-mediated endocytosis	12	6.37E-05
GO.005123	establishment of localization	50	0.000308
GO.004851	negative regulation of biological process	56	0.00031

GO.004852	negative regulation of cellular process	52	0.000896
GO.005089	response to stimulus	75	0.000896
GO.005117	localization	55	0.000896
GO.000681	transport	47	0.00108
GO.004428	small molecule metabolic process	35	0.00111
GO.005127	regulation of cellular component moveme	18	0.00349
GO.004428	small molecule biosynthetic process	13	0.00445
GO.003033	regulation of cell migration	16	0.00567
GO.003083	positive regulation of actin filament polym	6	0.00599
GO.004222	response to chemical	47	0.00795
GO.004424	cellular lipid catabolic process	8	0.00809
GO.001604	lipid catabolic process	10	0.00816
GO.001975	carboxylic acid metabolic process	18	0.00816
GO.004858	negative regulation of response to stimulu	24	0.00816
**Top 20 Donor D2 Enriched for upregulated proteins in CD14+ cells**			
GO.000695	response to stress	178	3.06E-16
GO.000237	immune system process	121	7.79E-15
GO.002290	respiratory electron transport chain	29	7.79E-15
GO.190257	single-organism localization	160	7.79E-15
GO.004533	cellular respiration	33	8.26E-15
GO.004476	single-organism transport	152	1.52E-14
GO.000695	defense response	96	2.73E-14
GO.004508	innate immune response	75	4.22E-14
GO.005123	establishment of localization	172	9.72E-14
GO.000681	transport	166	4.89E-13
GO.000695	immune response	89	4.89E-13
GO.004471	single-organism metabolic process	191	4.89E-13
GO.005164	cellular localization	120	4.89E-13
GO.005164	establishment of localization in cell	104	6.09E-12
GO.000268	regulation of immune system process	88	7.17E-12
GO.005117	localization	190	1.14E-11
GO.000699	organelle organization	143	1.25E-11
GO.000268	positive regulation of immune system pro	64	2.06E-11
GO.004476	single-organism cellular process	349	2.17E-11
GO.000914	nucleoside triphosphate metabolic proces	30	1.14E-10

GO ID	GO name	observed protein count	P-Value
**Top 20 Donor D3 Enriched for upregulated proteins in Immature dendritic cells**			
GO.000689	endocytosis	23	2.97E-07
GO.001619	vesicle-mediated transport	29	0.000222
GO.006500	regulation of biological quality	50	0.000502
GO.005117	localization	61	0.0021
GO.005149	regulation of cytoskeleton organization	15	0.0021
GO.005165	maintenance of location in cell	8	0.0021
GO.003295	regulation of actin cytoskeleton organizat	12	0.00314
GO.005123	maintenance of location	9	0.00359
GO.005123	establishment of localization	52	0.00375
GO.000810	protein localization	32	0.00428

GO.003303	macromolecule localization		36	0.00428
GO.000681	transport		50	0.00551
GO.003250	maintenance of protein location in cell		7	0.00551
GO.003304	regulation of organelle organization		24	0.00763
GO.004852	negative regulation of cellular process		54	0.00866
GO.005112	regulation of cellular component organiza		36	0.00866
GO.000806	regulation of actin polymerization or depo		8	0.00978
GO.003083	positive regulation of actin filament polym		6	0.00978
GO.001931	hexose metabolic process		9	0.0116
GO.003227	regulation of protein polymerization		8	0.0116
**Top 20 Donor D3 Enriched for upregulated proteins in CD14+ cells**				
GO.004533	cellular respiration		44	2.85E-24
GO.002290	respiratory electron transport chain		34	2.61E-19
GO.001598	energy derivation by oxidation of organic		49	2.46E-17
GO.005511	oxidation-reduction process		86	5.98E-17
GO.005164	cellular localization		133	3.40E-16
GO.000609	generation of precursor metabolites and e		52	3.47E-16
GO.005164	establishment of localization in cell		117	1.36E-15
GO.005123	establishment of localization		182	9.40E-15
GO.000681	transport		176	4.25E-14
GO.004428	small molecule metabolic process		129	4.25E-14
GO.190257	single-organism localization		163	4.25E-14
GO.004690	intracellular transport		94	5.10E-14
GO.000920	purine ribonucleoside triphosphate metab		33	5.24E-14
GO.000914	nucleoside triphosphate metabolic proces		35	5.30E-14
GO.003303	macromolecule localization		124	5.30E-14
GO.004476	single-organism transport		154	1.69E-13
GO.190258	single-organism intracellular transport		85	1.74E-13
GO.005117	localization		203	2.60E-13
GO.004603	ATP metabolic process		30	1.05E-12
GO.000810	protein localization		105	5.34E-12
