# Supplementary material for: Biogenesis of HLA Ligand Presentation in Immune Cells Upon Activation Reveals Changes in Peptide Length Preference
Source: Front Immunol. 2020 Aug 28;11:1981. doi: 10.3389/fimmu.2020.01981 (PMC7485268; doi:10.3389/fimmu.2020.01981)
Supplement: Supplementary Table 9 — GO enrichment of molecular functions upon DC maturation. GO enrichment of molecular functions was calculated with String (version 10.5). For this analysis, from each donor were used proteins found to be significantly upregulated (FDR = 1%, S0 = 3) upon maturation of immature dendritic cells in proteomics analysis. The top 20 enriched functions are reported. [file Data_Sheet_9.PDF]

Supplementary Table 9

GO ID	GO name	observed protein count	P-Value
**Top 20 Donor D1 enriched for upregulated proteins in matured dendritic cells**			
GO.0045087	innate immune response	63	2.91E-35
GO.0051607	defense response to virus	33	2.34E-32
GO.0051707	response to other organism	51	2.85E-30
GO.0009615	response to virus	35	8.45E-30
GO.0006952	defense response	67	1.17E-29
GO.0060337	type I interferon signaling pathway	24	2.28E-29
GO.0071357	cellular response to type I interferon	24	2.28E-29
GO.0002252	immune effector process	42	6.58E-29
GO.0006955	immune response	64	7.20E-29
GO.0098542	defense response to other organism	38	1.99E-27
GO.0034097	response to cytokine	45	1.35E-25
GO.0019221	cytokine-mediated signaling pathway	37	4.95E-25
GO.0034341	response to interferon-gamma	24	4.55E-24
GO.0002376	immune system process	68	3.02E-22
GO.0071346	cellular response to interferon-gamma	21	2.72E-21
GO.0071345	cellular response to cytokine stimulus	37	2.53E-20
GO.0043901	negative regulation of multi-organism pr	22	2.94E-18
GO.0060333	interferon-gamma-mediated signaling pa	17	5.75E-18
GO.0048525	negative regulation of viral process	18	1.12E-17
GO.0006950	response to stress	81	1.81E-17

GO ID	GO name	observed protein count	P-Value
**Top 20 Donor D4 enriched for upregulated proteins in matured dendritic cells**			
GO.0002252	immune effector process	58	1.80E-26
GO.0006952	defense response	97	4.00E-24
GO.0045087	innate immune response	79	4.00E-24
GO.0051707	response to other organism	67	4.10E-24
GO.0006950	response to stress	156	8.83E-23
GO.0051607	defense response to virus	35	8.83E-23
GO.0009615	response to virus	39	3.98E-21
GO.0060337	type I interferon signaling pathway	24	2.42E-20
GO.0071357	cellular response to type I interferon	24	2.42E-20
GO.0006955	immune response	86	6.20E-20
GO.0002376	immune system process	108	8.38E-20
GO.0034341	response to interferon-gamma	27	1.06E-18
GO.0098542	defense response to other organism	43	4.35E-18
GO.0034097	response to cytokine	56	9.12E-18
GO.0071346	cellular response to interferon-gamma	24	3.60E-17
GO.0010033	response to organic substance	115	3.12E-16
GO.0060333	interferon-gamma-mediated signaling pa	20	1.51E-15
GO.0002682	regulation of immune system process	79	2.77E-15
GO.0019221	cytokine-mediated signaling pathway	41	2.78E-15
GO.0051704	multi-organism process	105	5.33E-15
**GO ID**	**GO name**	**observed protein count**	**P-Value**
**Top Donor D4 Enriched for upregulated proteins in Immature dendritic cells**			
GO.0022613	ribonucleoprotein complex biogenesis	11	0.00189

GO.0034660	ncRNA metabolic process	12	0.00189
GO.0006364	rRNA processing	7	0.0156
GO.0006396	RNA processing	13	0.0471

GO ID	GO name	observed protein count	P-Value
**Top 20 Donor D2 enriched for upregulated proteins in matured dendritic cells**			
GO.0051607	defense response to virus	13	1.60E-14
GO.0045087	innate immune response	20	9.34E-14
GO.0051707	response to other organism	18	9.34E-14
GO.0060337	type I interferon signaling pathway	10	9.80E-14
GO.0071357	cellular response to type I interferon	10	9.80E-14
GO.0019221	cytokine-mediated signaling pathway	14	4.67E-12
GO.0002252	immune effector process	14	2.07E-11
GO.0006952	defense response	20	3.80E-11
GO.0034097	response to cytokine	15	1.29E-10
GO.0006955	immune response	19	1.34E-10
GO.0071345	cellular response to cytokine stimulus	13	4.53E-09
GO.0002376	immune system process	20	1.45E-08
GO.0045071	negative regulation of viral genome repli	6	1.93E-07
GO.0006950	response to stress	23	5.46E-07
GO.0034341	response to interferon-gamma	7	8.57E-07
GO.0051704	multi-organism process	18	5.43E-06
GO.0071346	cellular response to interferon-gamma	6	1.00E-05
GO.0001817	regulation of cytokine production	10	1.19E-05
GO.0007166	cell surface receptor signaling pathway	17	1.19E-05
GO.0071310	cellular response to organic substance	16	1.92E-05

GO ID	GO name	observed protein count	P-Value
**Top Donor D2 Enriched for upregulated proteins in Immature dendritic cells**			
GO.0000786	nucleosome	3	0.0362

GO ID	GO name	observed protein count	P-Value
**Top 20 Donor D3 Enriched for upregulated proteins in matured dendritic cells**			
GO.0045087	innate immune response	26	2.01E-20
GO.0051607	defense response to virus	16	1.28E-19
GO.0051707	response to other organism	22	2.57E-18
GO.0060337	type I interferon signaling pathway	12	2.01E-17
GO.0071357	cellular response to type I interferon	12	2.01E-17
GO.0019221	cytokine-mediated signaling pathway	18	2.77E-17
GO.0006952	defense response	26	4.32E-17
GO.0002252	immune effector process	18	1.81E-16
GO.0006955	immune response	24	2.85E-15
GO.0034097	response to cytokine	19	4.64E-15
GO.0048525	negative regulation of viral process	10	1.01E-12
GO.0045071	negative regulation of viral genome repli	8	3.85E-11
GO.1903901	negative regulation of viral life cycle	9	5.04E-11
GO.0050792	regulation of viral process	11	5.51E-11
GO.0071345	cellular response to cytokine stimulus	15	5.51E-11
GO.0051704	multi-organism process	24	1.82E-10
GO.0002376	immune system process	23	1.85E-10

GO.0034341	response to interferon-gamma	9	7.31E-10
GO.1903900	regulation of viral life cycle	10	8.84E-10
GO.0006950	response to stress	27	3.03E-09
**GO ID**	**GO name**	**observed protein count**	**P-Value**
**Donor D3 Enriched for upregulated proteins in Immature dendritic cells**			
GO.0008104	protein localization	22	0.0179
GO.0045184	establishment of protein localization	19	0.0179
