# Supplementary material for: Biogenesis of HLA Ligand Presentation in Immune Cells Upon Activation Reveals Changes in Peptide Length Preference
Source: Front Immunol. 2020 Aug 28;11:1981. doi: 10.3389/fimmu.2020.01981 (PMC7485268; doi:10.3389/fimmu.2020.01981)
Supplement: Supplementary Table 10 — Average peptide length across different HLA class I alleles of naive and activated immune cells (related to Figure 2A). For each donor, MS detected ligands (length 8–14) were separately assigned to each of the HLA alleles with NetMHCpan 4.0 and their average length was calculated. [file Data_Sheet_10.PDF]

Supplementary Table 10. Average peptide length across different HLA class I alleles of naive and activated immune cells (related to Figure 2A). For each donor, MS detected ligands (length 8-14) were separately assigned to each of the HLA alleles with NetMHCpan 4.0 and their average length was calculated.

**Cell type**	**HLA-A01:01**	**HLA-A02:01**	**HLA-A11:01**	**HLA-A26:08**	**HLA-A32:01**	**HLA-B08:01**	**HLA-B15:01**	**HLA-B27:05**	**HLA-B40:01**	**HLA-B44:02**	**HLA-B44:03**	**HLA-B51:01**
**CD14+**	9.62	9.08	9.57		9.12	8.84	9.17		9.27		9.46	8.99
**ImmDC**	9.82	9.08	9.61	9.19	9.16	8.87	9.2	9.47	9.41	9.49	9.6	8.98
**MaDC**	10.01	9.09	9.67	9.24	9.19	8.84	9.21	9.49	9.49	9.55	9.69	9
**CD8+**	9.69	9.06	9.58		9.1	8.86			9.27		9.52	
**CD8+ Activated**	9.96				9.19	8.88			9.58			
**CD4+**	9.62	9.07	9.55		9.1	8.86			9.26		9.5	
**CD4+ Activated**	9.95				9.2	8.89			9.57			
**CD19+**	9.74	9.07	9.61		9.11	8.88			9.39		9.57	
**CD19+ Activated**	9.97				9.18	8.88			9.52			
