# Supplementary material for: Biogenesis of HLA Ligand Presentation in Immune Cells Upon Activation Reveals Changes in Peptide Length Preference
Source: Front Immunol. 2020 Aug 28;11:1981. doi: 10.3389/fimmu.2020.01981 (PMC7485268; doi:10.3389/fimmu.2020.01981)
Supplement: Supplementary Table 11 — HLA class I peptides from synthetic peptides from Donor 5 to 7. [file Data_Sheet_11.PDF]

Supplementary Table 10. HLA class I peptides from synthetic peptides from Donor 5 to 7.

D5 HLAIp_ImmDC	D5 HLAIp_MaDC	D7 HLAIp_ImmDC	D7 HLAIp_MaDC	D6 HLAIp_ImmDC	D6 HLAIp_MaDC	Length	Mass	PEP	Score	MS/MS Count	Start position	End position	Sequence	Leading razor protein	Gene names	Protein names
23.09015	24.51779	23.60727	24.37245	NaN	NaN	9	1066.534	0.025639	91.041	1	207	215	FTITDQVPF	P40967-4	PMEL201	Melanocyte protein PMEL;M-alpha;M-beta
23.85614	NaN	NaN	NaN	NaN	NaN	13	1170.636	0.013	85.355	3	165	177	GPVSGLSIGTGRA	P40967-4	PMEL155	Melanocyte protein PMEL;M-alpha;M-beta
22.6435	NaN	NaN	NaN	NaN	NaN	14	1301.676	0.00081	104.82	2	165	178	GPVSGLSIGTGRAM	P40967-4	PMEL155	Melanocyte protein PMEL;M-alpha;M-beta
NaN	NaN	24.74489	26.94033	NaN	NaN	9	942.4705	0.12595	44.719	2	175	183	GRAMLGTHT	P40967-4	PMEL155	Melanocyte protein PMEL;M-alpha;M-beta
NaN	NaN	25.58236	28.19761	NaN	NaN	10	1073.511	0.027973	87.181	5	175	184	GRAMLGTHTM	P40967-4	PMEL155	Melanocyte protein PMEL;M-alpha;M-beta
NaN	NaN	23.3507	25.32485	NaN	NaN	12	1301.622	0.12205	65.395	1	175	186	GRAMLGTHTMEV	P40967-4	PMEL155	Melanocyte protein PMEL;M-alpha;M-beta
26.93146	26.43151	NaN	NaN	NaN	NaN	9	1043.62	0.000208	122.33	3	34	42	GVSRQLRTK	P40967-4	PMEL32	Melanocyte protein PMEL;M-alpha;M-beta
26.10368	26.09545	22.14482	25.06915	27.58828	21.44395	9	1115.507	0.024542	111.28	3	182	190	HTMEVTVYH	P40967-4	PMEL155	Melanocyte protein PMEL;M-alpha;M-beta
NaN	NaN	26.79365	28.21345	NaN	NaN	9	1004.518	0.014111	105.04	8	209	217	ITDQVPFSV	P40967-4	PMEL201	Melanocyte protein PMEL;M-alpha;M-beta
NaN	NaN	25.01325	26.9382	NaN	NaN	11	1226.652	0.009695	106.67	2	222	232	LRALDGGNKHF	P40967-4	PMEL201	Melanocyte protein PMEL;M-alpha;M-beta
NaN	NaN	27.2316	29.90297	NaN	NaN	9	1171.657	0.055618	66.568	2	39	47	LRTKAWNRQ	P40967-4	PMEL32	Melanocyte protein PMEL;M-alpha;M-beta
22.3894	25.47051	NaN	NaN	NaN	NaN	9	904.48	0.081758	63.565	1	170	178	LSIGTGRAM	P40967-4	PMEL155	Melanocyte protein PMEL;M-alpha;M-beta
NaN	NaN	NaN	26.27571	NaN	NaN	9	1017.462	0.01012	114.72	3	178	186	MLGTHTMEV	P40967-4	PMEL155	Melanocyte protein PMEL;M-alpha;M-beta
NaN	NaN	22.72972	24.63145	NaN	NaN	9	797.4647	0.028191	88.35	5	162	170	VLGGPVSGL	P40967-4	PMEL155	Melanocyte protein PMEL;M-alpha;M-beta
21.86306	26.46694	NaN	NaN	NaN	NaN	12	1372.713	0.076119	52.693	1	98	109	TPMEAELARRSL	P78358-2	CTAG1A	Cancer/testis antigen 1
NaN	NaN	23.85	25.20695	NaN	NaN	12	1293.715	0.064803	76.827	1	105	116	ARRSLAQDAPPL	P78358-2	CTAG1A	Cancer/testis antigen 1
NaN	NaN	24.40711	26.64306	NaN	NaN	11	1222.678	0.014511	97.431	2	106	116	RRSLAQDAPPL	P78358-2	CTAG1A	Cancer/testis antigen 1
NaN	NaN	NaN	NaN	24.5693	23.056	10	1211.67	0.070475	71.349	1	114	123	VDELAHFLLR	P43358	MAGEA4	Melanoma-associated antigen 4
